# *Tetrastigma hemsleyanum* (Sanyeqing) root extracts evoke S phase arrest while inhibiting the migration and invasion of human pancreatic cancer PANC-1 cells

**DOI:** 10.1186/s12906-024-04425-1

**Published:** 2024-03-27

**Authors:** Yifan Sun, Haiyan Qin, Chunchun Zhang, Jian Xu, Ting Zhang

**Affiliations:** 1 School of Medical Technology and Information Engineering, Zhejiang Chinese Medical University, Binwen Road, Binjiang District, Hangzhou, Zhejiang Province 310053 People’s Republic of China; 2 School of Pharmaceutical Sciences, Zhejiang Chinese Medical University, Hangzhou, 310053 People’s Republic of China; 3 Nanjing Healthnice Pharmaceutical Technology Co., Ltd CN, Nanjing, 210031 People’s Republic of China

**Keywords:** *Tetrastigma hemsleyanum*, GC-MS, PANC-1 cell, Migration, Invasion

## Abstract

**Background:**

Ethyl acetate extracts from *Tetrastigma hemsleyanum* (Sanyeqing) (EFT), a member of the Vitaceae plant family, have been shown to exhibit efficacy against a variety of cancers. In this light, our current study seeks to examine the mechanism of efficacy between EFT extracts and human pancreatic cancer PANC-1 cells.

**Methods:**

The chemical components of EFT were analyzed by gas chromatography–mass spectrometry. The cytotoxicity of EFT on PANC-1 cells was measured using an MTT assay. In order to investigate EFT induction of cell cycle arrest, changes in cell-cycle distribution were monitored by flow cytometry. Wound healing and transwell assays were employed to investigate whether migration and invasion of PANC-1 cells were inhibited by EFT. Relative protein expression was detected using Western blot.

**Results:**

GC-MS analysis of the chemical composition of EFT revealed that the majority of constituents were organic acids and their corresponding esters. EFT exhibits measurable cytotoxicity and inhibition of PANC-1 invasion. Growth inhibition was primarily attributed to downregulation of CDK2 which induces cell cycle arrest in the S-phase. Inhibition of metastasis is achieved through downregulation of mesenchymal-associated genes/activators, including ZEB1, N-cadherin, Vimentin, and Fibronectin. Meanwhile, the expression of E-cadherin was significantly increased by EFT treatment. Furthermore, downregulation of MMP-2 and MMP-9 were observed.

**Conclusion:**

Treatment of PANC-1 with EFT demonstrated measurable cytotoxic effects. Furthermore, EFT evoked S phase arrest while inhibiting the migration and invasion of PANC-1 cells. Additionally, EFT inhibited the epithelial to mesenchymal transition and MMPs expression in PANC-1 cells. This study serves to confirm the strong therapeutic potential of EFT while identifying the mechanisms of action.

**Supplementary Information:**

The online version contains supplementary material available at 10.1186/s12906-024-04425-1.

## Background

It is estimated that fatalities arising from pancreatic cancer will be second only to lung cancer by 2030. Overall 5-year survival rates for pancreatic cancer have remained unchanged for decades and patient prognosis remains poor despite efforts to better understand the tumor microenvironment [1–3]. Therefore, novel treatment strategies for pancreatic cancer are in dire need. Current chemotherapeutic regimens used in treating pancreatic cancer suffer from debilitating side effects which limit tolerable doses [4]. Furthermore, many therapies exacerbate the mutation of cancers to metastatic and drug resistant phenotypes. Yet, the majority of modern cancer therapeutics are derived from plants [5]. Concerning anti-tumor effects, previous studies have reported that various natural products can demonstrate anticancer efficacy through inhibition of cell growth [5, 6].

*Tetrastigma hemsleyanum* Diels et. Gilg (Sanyeqing) is a rare, endangered medicinal plant native to China and traditionally used in folk medicine to treat a variety of cancers. Extracts from its root tuber, stems, leaves or whole plant are proven to be effective against various types of cancer [6–10]. For example, flavonoids isolated from *Tetrastigma hemsleyanum* can suppress regulatory T cells preventing the development of lung cancer [10]. Additionally, polysaccharide from *Tetrastigma hemsleyanum* has been shown to enhance macrophage activation to inhibit the proliferation of breast cancer cells [9]. However, it is worth noting that most studies have mainly focused on extracting components from the stems and leaves of *Tetrastigma hemsleyanum*, or using different solvent systems like ethyl acetate fraction and water fraction [6–10]. Further research on root-derived components like the Ethyl acetate extract is necessary to fully understand their therapeutic potential and mechanisms. Therefore, the present study serves to investigate the possible mechanisms of ethyl acetate extracts from *Tetrastigma hemsleyanum* (EFT) cytotoxicity against PANC-1 such as cell cycle arrest and inhibition of metastasis.

## Materials and methods

### Antibodies and reagents

PANC-1 cells and BxPC-3 cells were purchased from the Stem Cell Bank, Chinese Academy of Sciences. hTERT-HPNE cells were purchased from the BeNa Culture Collection (Suzhou, China, BNCC338221). Rabbit monoclonal antibodies against E-cadherin (1:1000, ab40772), CDK2 (1:1000, ab32147), MMP-2 (1:1000, ab181286), MMP-9 (1:1000, ab76003) along with Rabbit polyclonal antibody against Fibronection (1:1000, ab2413) and Mouse monoclonal antibody against N-cadherin (1:1000, ab98952) were obtained from Abcam, England. Mouse monoclonal antibody against β-actin (1:1000, sc-47778) were obtained from Santa Cruz Biotechnology, USA. Goat anti-rabbit (1:1000, GAR0072) and Goat anti-mouse (1:1000, GAM0072) HRP conjugated secondary antibodies were obtained from MultiSciences Biotech, China. Rabbit monoclonal antibodies against ZEB1 (1:1000, #3396) and Vimentin (1:1000, #5741) were obtained from Cell Signaling Technology, USA. The BCA protein assay kit (P0011), 3-[4,5-dimethylthiazol-2-yl] -2,5-diphenyltetrazolium bromide (MTT) cell proliferation and cytotoxicity assay kit (C0009M) were obtained from Beyotime Biotech, China. Dulbecco’s modified Eagle medium (DMEM) was obtained from HyClone, USA. RPMI-1640 was obtained from Gibco, USA. Fetal bovine serum was obtained from Sijiqing Biotech, China. Polyvinylidene fluoride (PVDF) membrane was obtained from Millipore Life Science, USA.

### Preparation of extracts

*Tetrastigma hemsleyanum* roots were purchased from Pan’an in the Zhejiang Province of China (Pan’an Chunfu Traditional Chinese Medicine Rural Cooperatives, Lot.20151108), and authenticated by Dr. Shuili Zhang (College of Pharmaceutical Science, Zhejiang Chinese Medical University, Hangzhou, China). *Tetrastigma hemsleyanum* root was air dried for four days and dried in a hot air oven at 42 ℃. Then the dried plant was ground to powder form. Two kilograms of the powdered *Tetrastigma hemsleyanum* root (particle size < 400 μm) was refluxed in 75% ethanol three times, 2 h for each reflux. Three extractions were then combined and filtered. Subsequently, ethanol was removed using a rotary vacuum evaporator at 60 ℃ under reduced pressure. The ethanol extract was suspended in water. Then the solution was sequentially partitioned with different solvents, including petroleum ether, diethyl ether, ethyl acetate and n-butanol. Ethyl acetate extracts from *Tetrastigma hemsleyanum* (EFT) was concentrated using a rotary evaporator. A stock solution of EFT was dissolved in dimethyl sulfoxide (DMSO), stored at 4 ℃, and diluted to the desired concentration with DMEM before each experiment. The final concentration of DMSO was 0.1% (v/v) then filtered through a 0.22-µm micro-filtrate membrane. The negative control was treated with medium containing 0.1% DMSO. The same DMSO concentration was used for each drug treatment.

### Gas chromatography–mass spectrometry (GC–MS) analysis

GC–MS analysis (Thermo Fisher Scientific, USA) was performed to analyze EFT composition with HP-55% phenyl methyl siloxane capillary column (30 m * 0.25 mm * 0.25 μm). The carrier gas was helium at a flow rate of 1.0 mL/min with a split ratio of 50:1 (v/v). The temperature increased at a rate of 8 ℃ /min from 30 ℃ (hold 5 min) to 120 ℃, then at a rate of 20 ℃/min from 120 ℃ to 240 ℃ (kept 10 min). The injector temperature was maintained at 230 ℃. Data generated were processed with TraceFinder™ 4.1 software (Thermo Fisher Scientific) based on retention time, MS fragment ions and isotopic pattern. The phytochemicals were identified with the standard mass spectra in NIST Mass Spectral Library.

### Cell culture and cell proliferation assay

The BxPC-3 and PANC-1 human pancreatic cancer cell lines were cultured in RPMI-1640 culture medium and DMEM culture medium, respectively, containing 10% fetal bovine serum (FBS) in a humidified incubator with 5% CO_2_ at 37 ˚C. The Nonmalignant pancreas epithelial hTERT-HPNE cells were used to test the toxicity compared to pancreatic cancer cell. Cells were plated in 96-well plates (at a density of 5 × 10^3^ cells per well in 100 µL complete culture medium) (corning, USA), incubated overnight, and treated with different fractions of Ethyl acetate extracts from *Tetrastigma hemsleyanum* (EFT) at the concentrations of 0, 25, 50, 100, 200 and 400 µg/mL for 24 h (or treated with different EFT at the concentrations of 0, 25, 50, 100, 200 and 400 µg/mL for 12, 24, 48, and 72 h). After treatment, cells were washed twice with phosphate-buffered saline (PBS) and incubated with 10 µL MTT (5 mg/mL) and 80 µL DMEM at 37 ℃ for 4 h. Then the supernatant was replaced by 150 µL DMSO to dissolve formazan. The absorbance was measured at 570 nm by a Microplate reader. The viability of PANC-1 cells was compared with relation to the control group (0.1% DMSO). Each assay was performed in triplicate. The percentage of cell growth inhibition was calculated as follows:

$$\eqalign{& {\rm{Cell}}\,{\rm{viability}}\,{\rm{inhibitory}}\,{\rm{rate}}\,\left( {\rm{\% }} \right)\,{\rm{ = }} \cr & \left[ {{\rm{A570}}\,\left( {{\rm{control}}} \right)\,{\rm{ - A570}}\,\left( {{\rm{EFT}}} \right)} \right] \cr & {\rm{/A570}}\,\left( {{\rm{control}}} \right){\rm{ \times }}\,{\rm{100\% }} \cr}$$

### Flow cytometric analysis of cell cycle

PANC-1 cells cultured in 6-well plates at a density of 5 × 10^5^ cells/mL were treated with EFT (50, 100, and 200 µg/mL) or 0.1% DMSO for 24 h. After treatment, cells were collected by centrifugation at 1000 g for 5 min. The pellet was washed with twice with PBS, fixed by 1 mL of 70% cold ethanol and kept at − 20 ℃ overnight. The ethanol was removed by centrifugation at 1000 g for 5 min. Then cells were resuspended in PBS. Finally, cells were incubated in PI/RNase Staining Buffer (PBS containing 50 µg/mL PI and 100 µg/mL RNaseA) in the dark at 37 ℃ for 30 min and immediately analyzed by a flow cytometry (FC 500, Beckman, USA). MultiCycle software was used to analyze cell cycle data. Three independent experiments were performed in this analysis.

### Wound healing assay

PANC-1 cells were seeded in six-well plates. When the cells formed a confluent monolayer, wounds were produced by scratching the middle of the well with a 200-µL pipette tip. The culture medium of PANC-1 cells was supplemented with EFT (50, 100, and 200 µg/mL) or 0.1% DMSO for 24 h. 1000 µg/mL gemcitabine were used as a comparison. The initial wound photomicrographs were taken for comparison after migration was allowed for 24 h. The percentage of wound closure was measured by light microscopy (Olympus, Tokyo, Japan). Experiments were conducted in triplicate. The migration distance was calculated as follows:

Migration distance = Edge distance at 0 h - Edge distance at 24 h.

### Migration and invasion assays

PANC-1 cells were seeded at a density of 2 × 10^5^ cells/2 mL in each 35-mm culture dish. After 12 h, the culture medium were supplemented with EFT (50, 100, and 200 µg/mL) or 0.1% DMSO for 24 h to prepare for thee cell suspension, and 1000 µg/mL gemcitabine were also used as a comparison. Thereafter, 800 µL of DMEM containing 20% serum was added to the lower chamber (bottom of the 24-well plate) after preparing the cells, and 200 µL of serum-free cell suspension was added to the upper chamber, the cells were then cultured in an incubator for 24 h. The culture medium in both upper and lower chambers was discarded, and the cells in both upper and lower chambers were washed with PBS, fixed by methanol respectively. The chamber was removed, and the upper and lower chamber fixatives were blotted until dry. Thereafter, 800 µL and 200 µL 0.1% crystal violet were added to the lower chamber and upper chamber respectively, and incubated at room temperature for 30 min. After rinsing with PBS for 3 times, 800 µL and 200 µL 0.2% DAPI were added to the lower chamber and upper chamber respectively, and incubated at room temperature for 15 min. The cells were then gently rinsed and soaked several times with water; the chamber was removed, the upper chamber liquid was absorbed, and the cells on the membrane surface on the bottom of the upper chamber were carefully wiped off with a damp cotton swab. Turn the chamber upside down and let dry. The samples were photographed in five independent, 100 X magnification fields for each well using a microscope (Olympus, Tokyo, Japan). The cell invasion assay were performed similarly using BD Matrigel Invasion Chambers with 8 μm pore-size PET membrane (BD BioCoat, 354480). Experiments were repeated in triplicate for each experiment.

### Western blotting

PANC-1 cells were seeded in 3.5 cm petri dishes (1 × 10^6^ cells/mL). Then, PANC-1 cells were treated with 50, 100 µg/mL EFT for 24 h and 200 µg/mL EFT for 12, 24, 48 h (0.1% DMSO as a control). The total protein of treated cells was extracted with radio-immunoprecipitation assay lysis buffer (1% Triton X-100, 0.1% sodium dodecyl sulfate (SDS), and 1% sodium deoxycholate). Supernatants were collected and protein concentrations determined using a BCA protein assay kit. The lysates were boiled for 5 min at 100 ℃. Equal amounts of total protein were separated by 8–10% sodium dodecyl sulfate-polyacrylamide gel electrophoresis (SDS-PAGE), transferred to PVDF, blocked with 5% non-fatty milk in TBS-Tween buffer (pH 7.5, 0.12 M Tris-base, 1.5 M NaCl, 0.1% Tween-20) for 2 h at room temperature, incubated with the corresponding primary antibodies separately overnight at 4 ℃ and then incubated with HRP conjugated secondary antibodies (Goat anti-rabbit or Goat anti-mouse) at room temperature in the dark for 2 h. Detection was performed using an ECL system. Protein quantification was normalized to β-actin expression. ImageJ software was used to quantify band intensities.

### Statistical analysis

Statistical comparisons were performed with the SPSS 17.0 software. The data are presented as the mean ± standard deviation from at least three independent experiments. Differences were analyzed using one-way analysis of variance (ANOVA) (three or more groups) or independent t-test (two groups). *P* < 0.05 was considered statistically significant.

## Results

### Chemical analysis of EFT composition by GC–MS

**Fig. 1:**
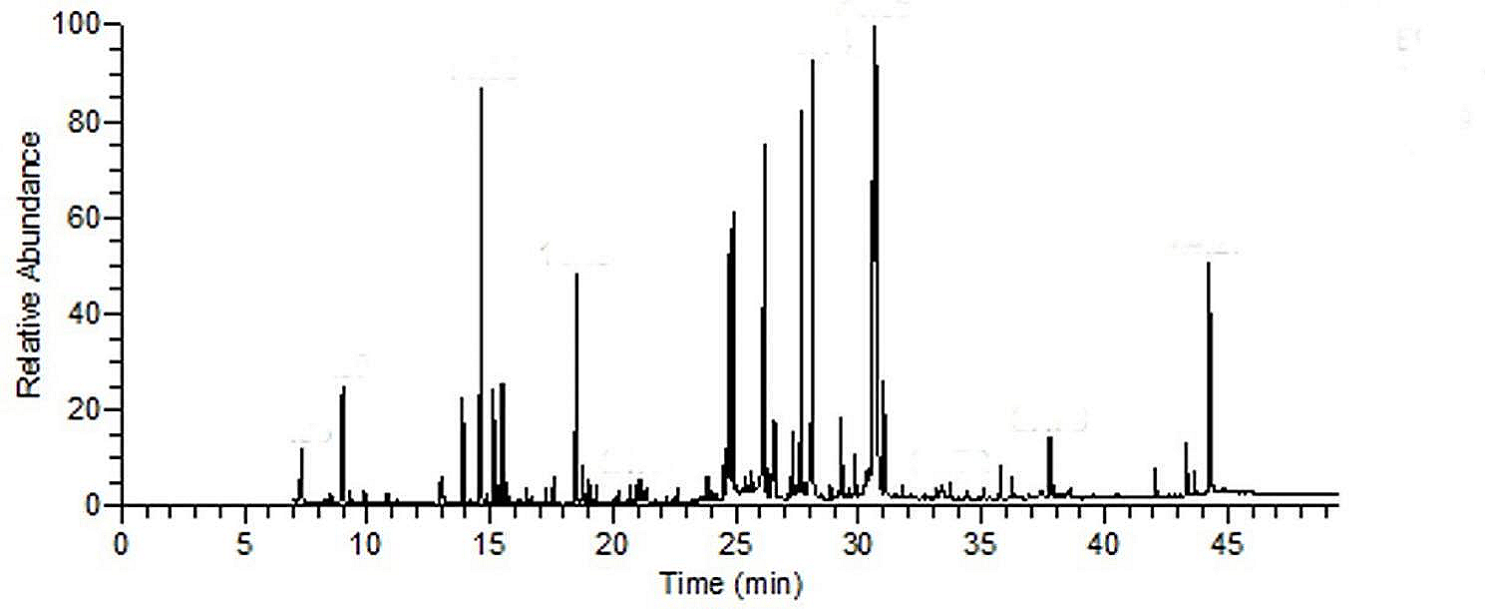
GC–MS analysis of EFT composition

EFT composition was analyzed by GC–MS and different components were identified by comparing their retention times (RIs) with literature values and their mass spectrum with the data library (Fig. 1). As shown in Table 1, a total 36 components were identified, representing 93.20% of the total compounds. The major constituents were succinic acid (10.56%), benzoic acid, 2-borono-4,5-dimethoxy-, 1-methyl ester (8.65%), palmitic acid (12.02%), linoleic acid (14.37%), and 9,12,15-all-cis-Octadecatrienoic acid (9.46%).

**Table 1:** Chemical constituents of EFT analyzed by GC–MS

Peak	Rt (min)	Molecular formula	Molecular weight	Compound	Area (%)
1	8.53	C_9_H_16_O	140	3,3,5-Trimethylcyclohexanone	0.74
2	8.99	C_3_H_6_O_3_	234	Lactic acid	1.42
3	13.07	C_4_H_10_O_3_	250	Diethylene glycol	0.25
4	13.88	C_3_H_8_O_3_	308	Glycerol	0.89
5	14.63	C_4_H_6_O_4_	262	Succinic acid	10.56
6	15.14	C_3_H_6_O_4_	322	2,3-dihydroxy-Propanoic acid	1.04
7	15.5	C_11_H_20_O_3_	200	5-Keto-2,2-dimethylheptanoic acid, ethyl(ester)	1.20
8	15.69	C_4_H_10_O_3_	322	1,2,4-Butanetriol	0.26
9	18.77	C_7_H_6_O_3_	282	Salicylic acid	3.12
10	24.48	C_8_H_12_O_7_	436	1,3-Dioxolane-4,5-dicarboxylicacid1,3-Dioxolane-4,5-dicarboxylicacid, 2-ethoxy-2-methyl-, (4R,5R)-	2.35
11	24.68	C_12_H_10_O_5_	438	Armillarisin A	3.28
12	24.82	C_6_H_10_O_7_	554	D-Glucuronic acid	3.31
13	24.9	C_6_H_12_O_6_	540	Dextrose	3.81
14	25.68	C_11_H_21_BO_6_	404	2-(1,3-Dioxolan-2-on-4-yl)-1-ethylboronic acid pinacol cyclic ester	0.24
15	26.48	C_9_H_8_O_3_	308	3-Phenylpyruvic acid	4.52
16	26.57	C_3_H_4_O_4_	320	Malonic acid	0.81
17	27.22	C_18_H_36_O_2_	284	Hexadecanoic acid, ethyl ester	0.24
18	27.77	C_10_H_13_BO_6_	420	Benzoic acid, 2-borono-4,5-dimethoxy-, 1-methyl ester	8.65
19	28.13	C_16_H_32_O_2_	328	Palmitic acid	12.02
20	28.85	C_17_H_36_	240	Tetradecane, 2,6,10-trimethyl	0.45
21	29.57	C_17_H_34_O_2_	342	Methyl hexadecanoate	0.72
22	29.81	C_20_H_36_O_2_	308	9,12-Octadecadienoic acid, ethyl ester	0.44
23	29.9	C_21_H_36_O_4_	352	9,12,15-Octadecatrienoicacid, 2,3-dihydroxypropyl ester	0.18
24	30.65	C_18_H_32_O_2_	352	Linoleic acid	14.37
25	30.74	C_18_H_30_O_2_	350	9,12,15-all-cis-Octadecatrienoic acid	9.46
26	31.02	C_18_H_36_O_2_	356	Stearic acid	1.28
27	33.38	C_23_H_46_O_2_S	382	11-Eicosenoic acid, trimethylsilyl ester	0.30
28	33.72	C_20_H_40_O_2_	384	Eicosanoic acid	0.20
29	35.11	C_16_H_22_O_4_	278	1,2-Benzenedicarboxylic acid, mono(2-ethylhexyl) ester	0.34
30	35.75	C_19_H_38_O_4_	474	Hexadecanoic acid,(2 S)-2,3-dihydroxypropyl ester	0.44
31	36.23	C_22_H_44_O_2_	412	Docosanoic acid	0.30
32	37.78	C_21_H_40_O_4_	500	9-Octadecenoic acid (Z)-, ester with 1,2,3-propanetriol	0.83
33	42.08	C_19_H_28_N_4_O_5_	392	4-Azido-2-nitrobutyric acid, 2,6-di-t-butyl-4-methoxyphenyl ester	0.35
34	43.34	C_28_H_48_O	400	Campesterol	0.70
35	43.65	C_29_H_48_O	484	Stigmasterol	0.35
36	44.27	C_29_H_50_O	486	beta-Sitosterol	3.78

### Cell proliferation cytotoxicity assay

**Fig. 2:**
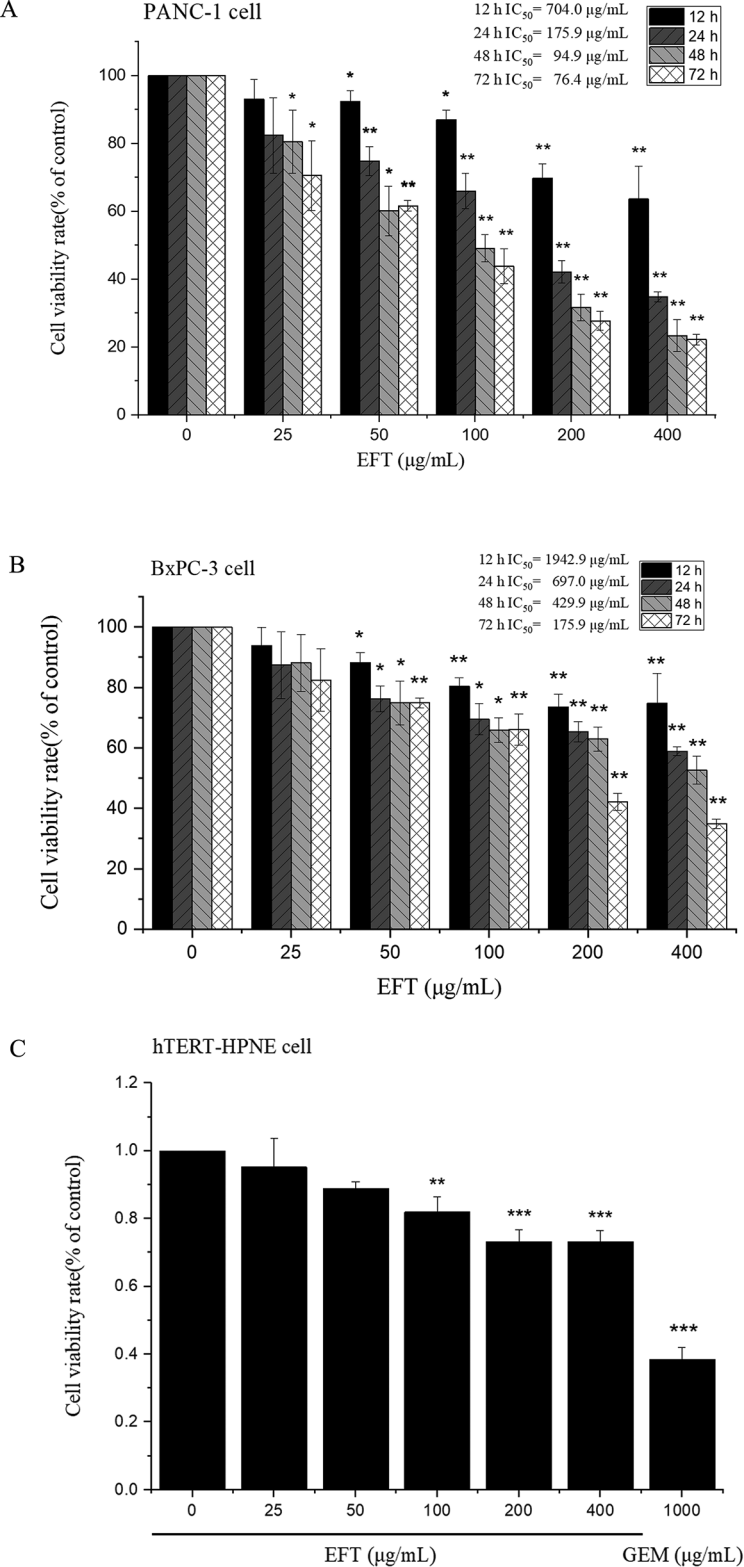
EFT inhibits cell proliferation in BxPC-3 and PANC-1 cells. PANC-1 cell (**A**) and BxPC-3 cell (**B**) viability inhibitory rate determined by MTT after incubation for 12 h, 24 h 48 h, and 72 h. (**C**) hTERT-HPNE cells were used as normal control. Data represent the mean ± SD of three replicates and three independent experiments. *P < 0.05, **P < 0.01, ***P < 0.001, compared with control (0.1% DMSO only)

To determine the efficacy of EFT against PANC-1 cells and BxPC-3 cells, an MTT assay was performed after incubating cells with the control (DMSO only), 25, 50, 100, 200, 400 µg/mL of EFT for 12, 24, 48 and 72 h. Cell viability and inhibitory rates were determined as seen from Fig. 2A and B. At the same time, in order to verify whether EFT have toxic effects on normal cells, we also used hTERT-HPNE cells for MTT experiment (Fig. 2C). Gemcitabine (1000 µg/mL) was determined as the positive control (Fig. S1). At a very low dose (25 µg/mL), EFT showed no significant difference with the control cells (*P *> 0.05) in both PANC-1 and BxPC-3 cells. The cell proliferation was inhibited in PANC-1 and BxPC-3 cells following treatment with 50 µg/mL EFT compared to the control (*P* < 0.05). The inhibitory effect was also found to increase in a time-dependent manner, with 72 h incubation demonstrating greater inhibition than earlier time points in PANC-1 and BxPC-3 cells (*P* < 0.05). Only 100 µg/mL EFT has a certain toxic effect on hTERT-HPNE cells, while 400 µg/mL EFT has a much lower toxicity to hTERT-HPNE cells than 1000 µg/mL gemcitabine (Fig. 2C and S2), indicating that EFT has limited damage to normal cells. The reduction in cell viability became constant at 48–72 h, the 50% inhibitory concentration of EFT (IC_50_) at 72 h were 76.4 µg/mL and 175.9 µg/mL respectively in PANC-1 and BxPC-3 cells determined using SPSS 17.0 software (Fig. 2A and B, Table S2-S3). Since EFT had a significantly higher inhibitory effect on PANC-1 cells than BXPC-3 cells, PANC-1 cell lines were used in subsequent experiments.

### Effects of EFT on cell cycle distribution in PANC-1 cells

**Fig. 3:**
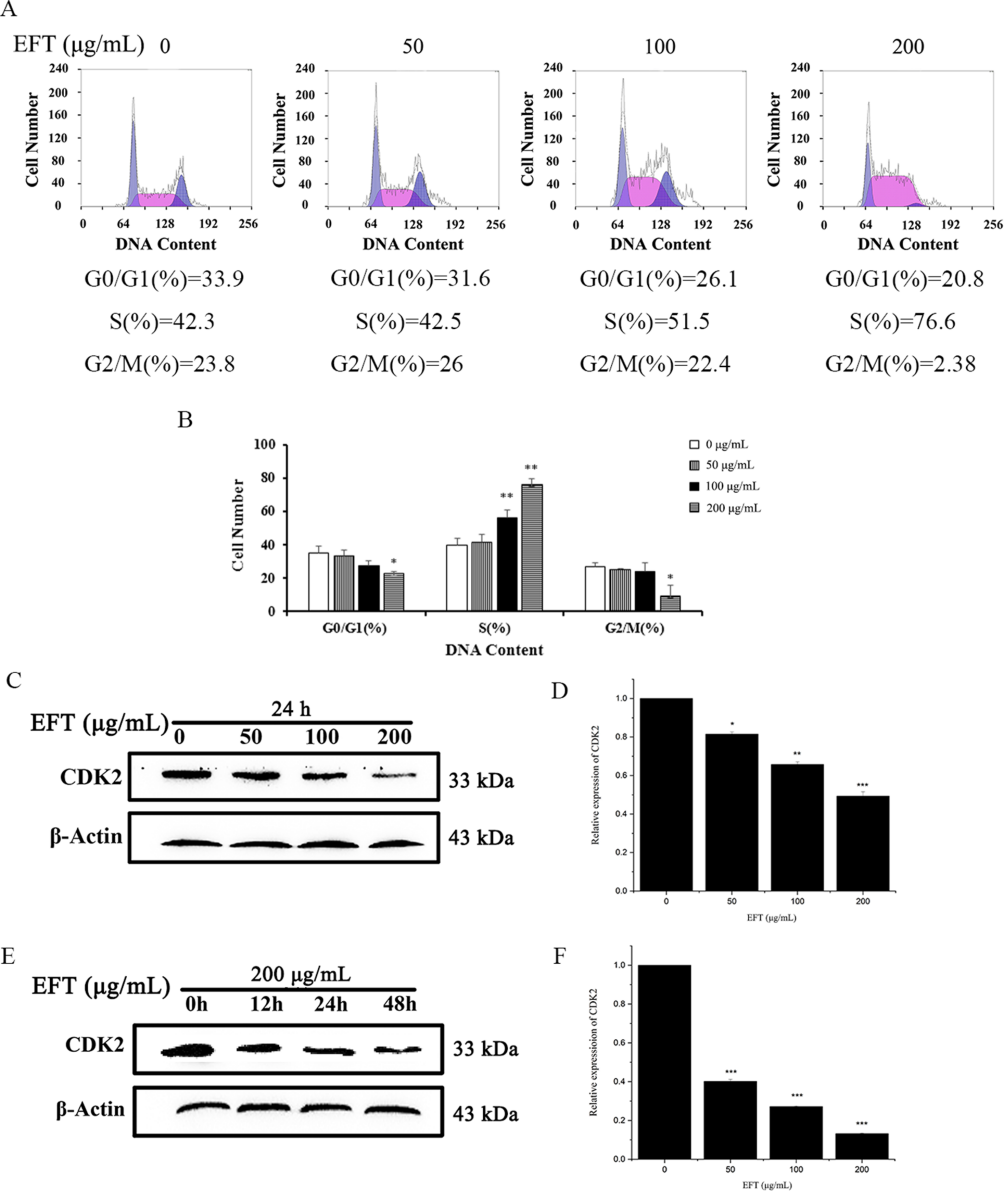
Effects of EFT on the cell-cycle in PANC-1 cells. (**A**) Cells were exposed to different concentrations of EFT for 24 h (0.1% DMSO as a control). After treatment, cells were harvested and subject to flow cytometry to assess the cell cycle distribution. At least 10,000 cells were analyzed per sample. (**B**) The distribution of cells in each phase of the cell cycle after treatment with 50, 100 and 200 µg/mL EFT (0.1% DMSO as a control). All tests were performed in triplicate and presented as mean ± standard deviation. (**C** and **E**) PANC-1 cells were treated with 50, 100 µg/mL EFT for 24 h and 200 µg/mL EFT for 12, 24, 48 h (0.1% DMSO as a control). The total protein of treated cells was determined. Western blot analysis of the effect of EFT on protein levels of CDK2 in PANC-1 cells. (**D** and **F**) Quantification of CDK2 ratios. *P < 0.05, **P < 0.01, ***P < 0.001, compared with control (0.1% DMSO only)

To determine whether EFT induces cell cycle arrest, the distribution of cells in various phases of the cell cycle was determined by flow cytometry. It was found that treatment of PANC-1 cells with EFT at 50, 100 and 200 µg/mL for 24 h induced cell cycle arrest (Fig. 3A). The number of PANC-1 cells at S stage after EFT treatment of 100 and 200 µg/mL was (56.4 ± 4.33) % and (75.8 ± 3.57) %, respectively, which was significantly higher (*P* < 0.01) than that of the control group (39.9 ± 3.79) % (Fig. 3B, Table S1). These results demonstrated cell cycle arrest in the S phase under treatment of EFT. Subsequently, the levels of CDK2 were determined by western blot analysis. Results showed decreased expression of CDK2 compared with the control. (Fig. 3C and D). A course experiment (0, 12, 24, 48 h) with the maximum dose of 200 µg/mL also showed a remarkable reduction in CDK2 expression (*P* < 0.01). (Fig. 3E and F).

### EFT inhibited cell migration and invasion

**Fig. 4:**
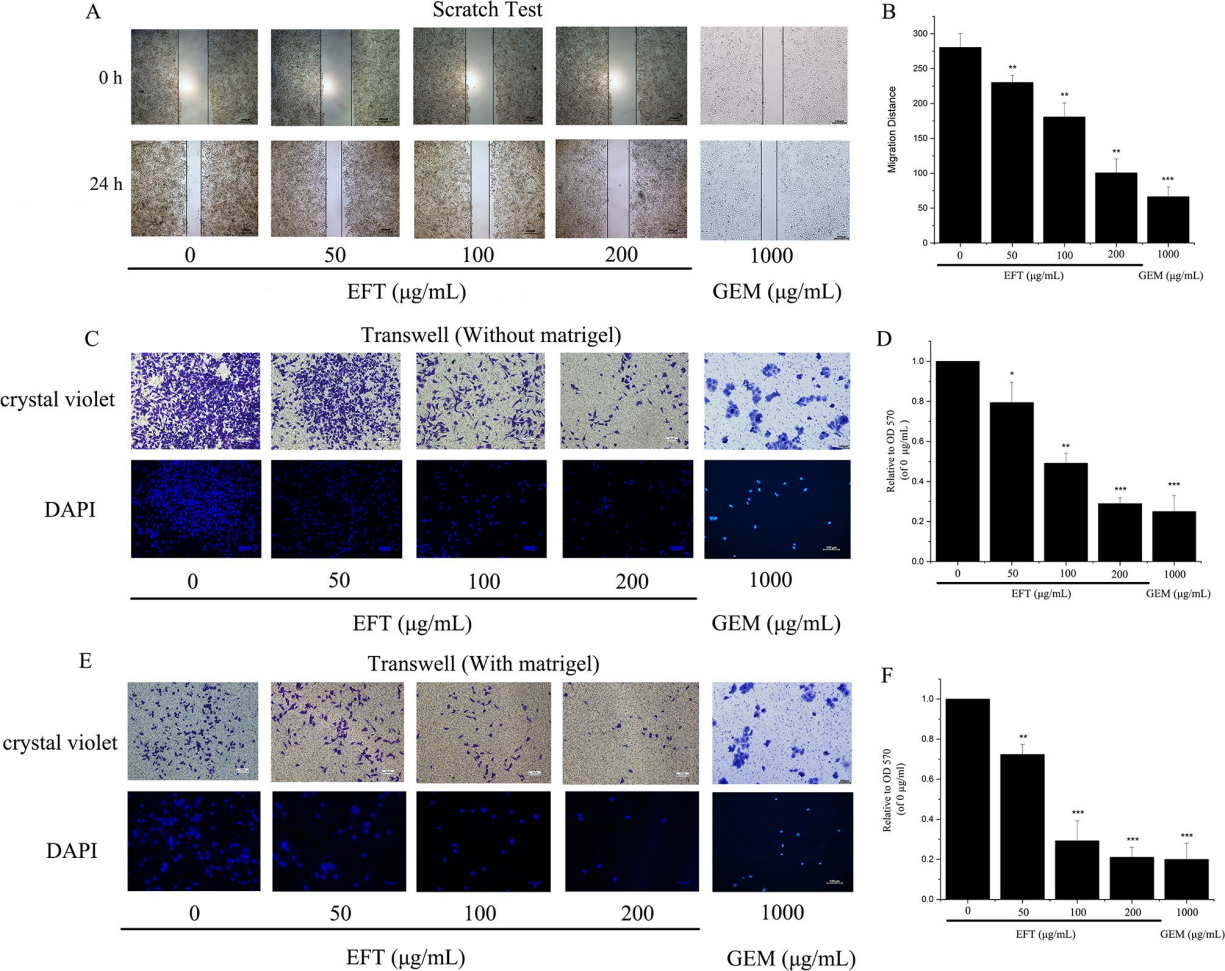
Effects of EFT on migration and invasion in PANC-1 cells. (**A**) The combined effects of migration and proliferation of EFT-treated PANC-1 cells were measured by a wound healing assay (magnification, ×40). (**B**) The migration distance was significantly decreased by EFT treatment. (**C**) Cells were treated with indicated concentrations of EFT for 24 h. The representative images of migrating cells at the bottom of the membrane stained with crystal violet and DAPI were visualized (magnification, ×100). (**E**) Cells were incubated with indicated concentrations of EFT for 24 h. Photographs illustrate cell invasion through the polycarbonate membrane stained by DAPI and crystal violet (magnification, ×100). (**D** and **F**) The stained cells were eluted by 33% glacial acetic acid for 20 min. The elution was measured at 570 nm to obtain the OD 570 values, which are normalized to that of the control. *P < 0.05, **P < 0.01, ***P<0.001, compared with control (0.1% DMSO only). Gemcitabine (1000 µg/mL) were used as the positive control

To analyze how the migration and proliferation of PANC-1 cells occurred, a wound healing assay was employed. As shown in Fig. 4A. EFT treatment significantly reduced the migration distance of PANC-1, which was (281.36 ± 9.12) µm in the control group and (101.72 ± 8.86) µm after 200 µg/mL EFT treatment (Fig. 4B, *P* < 0.01). In conjunction, transwell assays were conducted to examine whether migration could be inhibited by EFT. As shown in Fig. 4C and E, fewer cells were found to infiltrate the membranes with or without matrigel under the treatment of EFT, compared with untreated groups (*P* < 0.05) (Fig. 4D and F). The inhibitory effect of 200 µg/mL EFT on the migration and invasion of PANC-1 cells was similar to that of gemcitabine group, indicating that EFT can be potential alternative for gemcitabine therapy.

### EFT inhibited mesenchymal properties and MMPs in PANC-1 cells

**Fig. 5:**
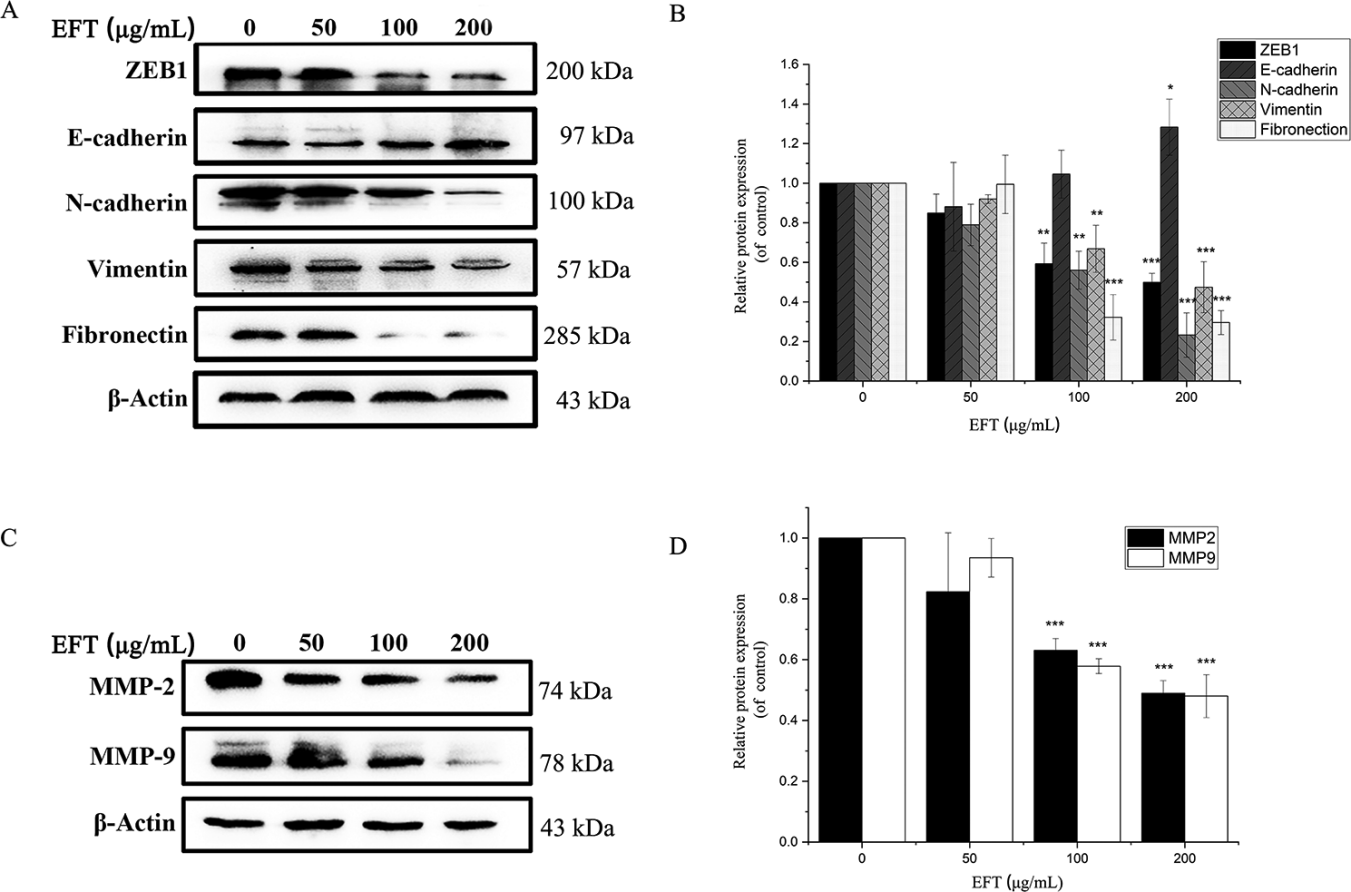
EFT inhibited mesenchymal properties and MMPs in PANC-1 cells. (**A** and **C**) EFT significantly decreased the expression of ZEB1, N-cadherin, Vimentin and Fibronectin as well as MMP-2 and MMP-9. Meanwhile,  E-cadherin was significantly increased by EFT treatment. (**B** and **D**) Total protein expression ratio was quantified against β-actin. All tests were performed in triplicate and presented as mean ± SD. *P < 0.05, **P < 0.01, ***P<0.001, compared with control (0.1% DMSO only)

Considering that the mesenchymal transition is closely related to migration and invasion of cancer cells, key molecular markers of Epithelial-Mesenchymal Transition (EMT) were observed before and after EFT treatment. Cells were incubated with EFT at concentrations of 0, 50,100, and 200 µg/mL before analysis. It was shown that EFT significantly decreased the expression of mesenchymal-associated genes/activators, including ZEB1, N-cadherin, Vimentin and Fibronectin. Meanwhile, the expression of E-cadherin was significantly increased by EFT treatment (Fig. 5A and B, *P* < 0.05). Two key enzymes of Matrix metalloproteinases (MMPs) family (MMP-2 and MMP-9) were also evaluated after EFT treatment. Fig. 5C and D illustrate a reduced expression of MMP-2 and MMP-9 following EFT incubation in a dose-dependent manner (*P* < 0.05). This result, however, reflects a change in the total expression of MMPs, further study should be involved to determine the changes of active MMPs. Yet, these results suggest that EFT is capable of inducing a pronounced inhibitory effect on the mesenchymal transition.

## Discussion

Pancreatic cancer is a leading cause of morbidity and mortality in both developed and developing countries [11, 12]. Characterized by its aggressive biological nature and strong resistance against chemotherapeutic treatment or radiation therapy, the 5-year survival rate of pancreatic cancer has shown little improvement in the past decade at < 5% survival [1, 3, 11–13]. Because chemotherapy and radiation therapy produce detrimental side effects, an urgent need for treatment strategies has arisen for pancreatic cancer. Recently, traditional Chinese medicines have garnered increasing attention due to their potential as anti-tumor agents. The majority of traditional Chinese medicines are derived from plant species, which have been used against cancer for centuries [5, 14, 15]. Yet, mechanisms underlying their efficacy remain unclear. Current research suggests that the mechanism of action occurs through cell cycle arrest, induction of apoptosis and inhibition of migration [8, 15–17]. Previous studies have shown that an ethyl acetate fraction of *Tetrastigma hemsleyanum* (EFT) can cause cell cycle arrest and induce apoptosis in human hepatoma HepG2 cells [7]. In this study, the underlying antitumor mechanisms were profiled in PANC-1 human pancreatic cancer cells treated with EFT.

Cell cycle control describes the regulatory process of cell growth that is dictated by coordinated interactions of various cyclins and their respective cyclin-dependent kinases (CDKs) [18, 19]. Cyclins and CDKs together form various checkpoints that can either usher or inhibit progression of a cell through the cell cycle. If the key steps at each regulatory phase of the cell cycle are completed, cyclin/CDK interactions allow progression into the next phase of the cycle [18–20]. However, prolonged arrest at a particular checkpoint can occur due to genetic instability or mutation; both key contributors to cancer development [20–22]. Not surprisingly, a variety of anticancer agents arrest the cell cycle at particular checkpoints to induce apoptosis in cancer cells [16, 21]. As seen from Fig. 3, S phase arrest was induced by EFT in a dose-dependent manner through downregulation of CDK2. This result indicates that the mechanism of EFT cancer therapy is, in part, due to the downregulation of CDK2 that induces cell cycle arrest and subsequent apoptosis. Yet, the mechanism of EFT is not solely limited to apoptosis. EFT has also shown potential to prevent migration and invasion of PANC-1 cells in a dose-dependent manner in Fig. 4. Since pancreatic cancer is characterized by excessive migration and invasion, methods to specifically inhibit or reverse these malignant features are crucial in both basic and preclinical research [22–24]. Largely, EFT’s anti-metastatic properties can largely be related to the epithelial to mesenchymal transition (EMT) and matrix metalloproteinases (MMPs).

EMT is a biological process where cells lose epithelial characteristics and obtain a mesenchymal phenotype, which improves cell motility by decreasing cell-cell adhesion [24]. Many studies point to the activation EMT as the critical mechanism for the acquisition of malignant phenotypes in epithelial cancer cells [24–27]. EMT is based on the down-regulation of epithelial markers such as E-cadherin while mesenchymal markers, such as ZEB1, Vimentin, N-cadherin and Fibronectin, are up-regulated [28]. Therefore, EMT or mesenchymal properties are an ideal target for cancer therapy.

MMPs are a family of zinc-dependent endopeptidases essential for extracellular matrix degradation [29]. Among all MMPs, MMP-2 and MMP-9 are key to basement membrane type IV collagen degradation during cancer progression. Particularly MMP-2 and MMP-9 are well known for promoting tumor migration and invasion [30, 31]. Aside from direct regulation of basement membrane degradation, cross talk between MMPs and EMT can regulate the invasion or migration of cancer cells. It has been demonstrated that overexpression of MMP-2 or MMP-9 led to induction of EMT in breast cancer [32]. Yet, a variety of EMT processes strengthen MMP expression in return, suggesting an intricate positive feedback loop between MMPs and EMT that synergistically contributes to migration and invasion in malignant tumors.

Therefore, strategies inhibiting both EMT and MMPs are an efficient approach to cancer therapy. As seen from Fig. 5, downregulation of mesenchymal markers such as ZEB1, Vimentin, N-cadherin and Fibronectin and upregulation of epithelial markers such as E-cadherin demonstrate the reversal of the metastatic EMT phenotype induced by EFT. Furthermore, downregulation of MMP-2 and MMP-9 were also identified after EFT treatment. These results suggest that EFT interrupts the positive feedback loop between EMT and MMPs by downregulating key cytokines known for EMT while also downregulating MMPs. These results further contribute to the anti-invasion properties found of EFT against PANC-1 human pancreatic cancer cells.

In summary, pancreatic cancer remains one of the most lethal cancers with 5% of 5-year survival rate. Its poor prognosis is mainly attributed to the nature of the cancer which is both drug resistant and highly metastatic. For treatment of pancreatic cancer, EFT has been identified as a potent therapeutic. As seen from Table 1, GC - MS analysis has revealed that the chemical constituents of EFT are primarily organic acids and their esters, among which succinic acid (10.56%) [33, 34] and benzoic acid (8.65%) had antioxidant, anti-inflammatory effects and had anti-tumor potential. Fatty acids [35–37] including linoleic acid (14.37%) and 9,12,15-all-cis-Octadecatrienoic acid (9.46%) have shown activity in cancer prevention and treatment. In recent years, a number of studies have shown that palmitic acid has become a promising antineoplastic agent with demonstrated effects on various malignancies including stomach cancer, liver cancer, cervical cancer, breast cancer and colorectal cancer [38–43]. The anti-tumor effects include inducing apoptosis of tumor cells, inhibiting proliferation of tumor cells, inhibiting metastasis and invasion, increasing sensitivity to chemotherapy, and improving immune function. Our findings suggest that EFT contains high levels of palmitic acid (12.02%) and may therefore have the same potential against tumors.

**Fig. 6:**
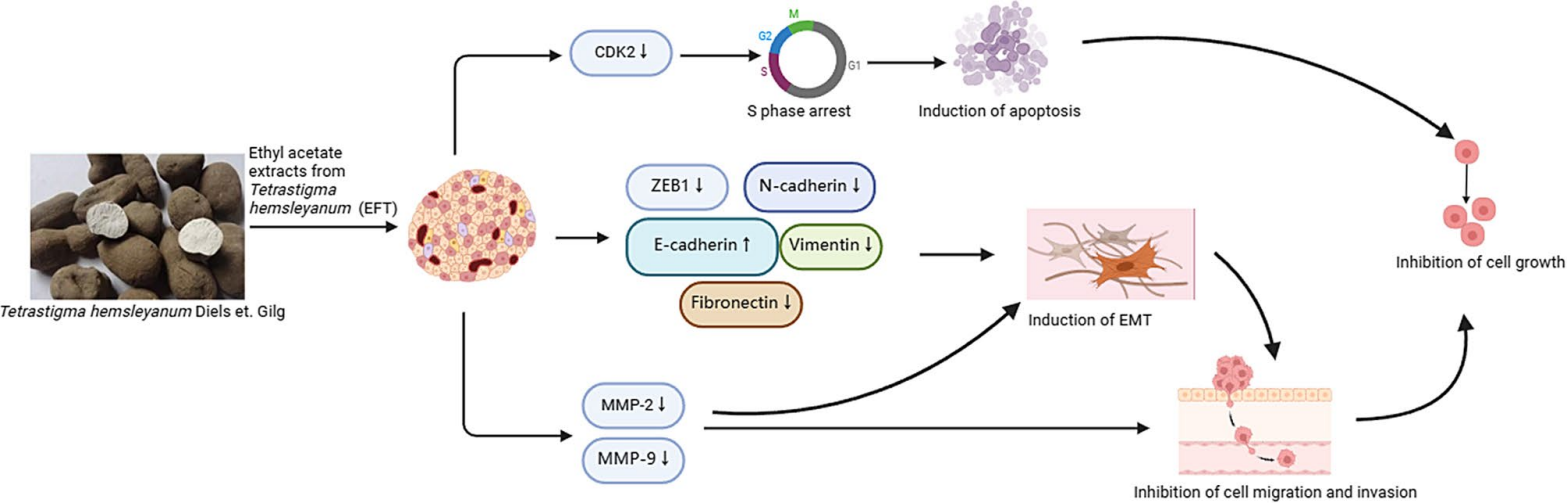
The proposed mechanism of EFT-induced S phase cell cycle arrest, inhibition of migration and invasion of PANC-1 cells

While EFT has shown potent efficacy against PANC-1 cells in vitro, its mechanism of efficacy is poorly understood. Studies performed herein demonstrated that EFT demonstrates significant cytotoxicity against PANC-1 through a cytotoxicity assay. Furthermore, EFT was found to inhibit PANC-1 migration through a wound healing assay. It was determined that EFT inhibits progression of PANC-1 through a collection of key properties. Firstly, EFT halts PANC-1 growth by inducing S-phase cell cycle arrest through downregulation of CDK2. Secondly, EFT tackles the issue of PANC-1 invasion and metastasis through a two-pronged approach. By increasing expression of cell-cell adhesion molecules, EFT substantially reverses the EMT, a key contributor to metastasis. Furthermore, EFT acts to downregulate MMP-2 and MMP-9 which are primarily known for inducing basement membrane degradation. By strengthening the basement membrane and promoting cell-cell adhesion, EFT interrupts the positive feedback loop between EMT and MMPs that are known contributors to the metastasis of PANC-1 cells. Based on the results of this study, the mechanisms by which EFT affects PANC-1 cells are summarized in Fig. 6.

## Conclusion

This study confirmed the anti-cancer properties of EFT on human pancreatic cancer PANC-1 cells. EFT exhibits efficacy through multiple avenues; such as inhibition of cell growth, inhibition cell invasion, cell cycle arrest, inhibition of mesenchymal properties and MMPs. In conclusion, these results affirm that *Tetrastigma hemsleyanum* serves as a valuable therapeutic for the treatment of pancreatic cancer.

### Electronic supplementary material

Below is the link to the electronic supplementary material.

Supplementary Material 1

Supplementary Material 2

Supplementary Material 3

## Data Availability

The data will be accessible by contacting the corresponding author of this study.
