# Supplementary material for: Tetrastigma hemsleyanum (Sanyeqing) root extracts evoke S phase arrest while inhibiting the migration and invasion of human pancreatic cancer PANC-1 cells
Source: BMC Complement Med Ther. 2024 Mar 27;24:133. doi: 10.1186/s12906-024-04425-1 (PMC10967071; doi:10.1186/s12906-024-04425-1)
Supplement: Supplementary file 1 — Supplementary Material 1 [file 12906_2024_4425_MOESM1_ESM.doc]

**Thermo Focus GC/DSQ two MS library search**

Data File: sanyeqing_TMS02

Original Data Path: sanyeqing_TMS02.RAW

Current Data Path: E:\DATA2016\

Sample Type: Unknown

Sample ID: 1

Sample Name:

Operator: DSQ

Acquisition Date: 10/12/16 14:02:20

Run Time (min): 42.53

Comments:

Vial: 1

Injection Volume (µl): 10.00

Scans: 11774

Low Mass (m/z): 45.00

High Mass (m/z): 600.00

Sample Volume (µl): 0.00

Sample Weight: 0.00

ISTD Amount: 0.000

Calibration Level:

Dilution Factor: 1.00

Instrument Method: F:\DATA2007\main\sanyeqing20161012TMS.meth

Processing Method: None

Calibration File: None

Revision: 1.4 SR1

Barcode: N/A

Barcode Status: Barcode Not Read

| Compound Structure | Delta | Hit Spectrum |
| --- | --- | --- |
|  |  |  |
|  |  |  |
|  |  |  |

| RT | Name | Library | Cas # | Molecular Formula | MolecularWeight | Probability | SI | RSI |
| --- | --- | --- | --- | --- | --- | --- | --- | --- |
| 7.29 | Tris(trimethylsilyl)borate | MAINLIB | 4325-85-3 | C9H27BO3Si3 | 278 | 59.97 | 641 | 710 |
| 7.29 | Mercaptoacetic acid, bis(trimethylsilyl)- | MAINLIB | 6398-62-5 | C8H20O2SSi2 | 236 | 13.00 | 597 | 727 |
| 7.29 | Trisiloxane, octamethyl- | MAINLIB | 107-51-7 | C8H24O2Si3 | 236 | 3.82 | 570 | 686 |

| Compound Structure | Delta | Hit Spectrum |
| --- | --- | --- |
|  |  |  |
|  |  |  |
|  |  |  |

| RT | Name | Library | Cas # | Molecular Formula | MolecularWeight | Probability | SI | RSI |
| --- | --- | --- | --- | --- | --- | --- | --- | --- |
| 7.35 | Tris(trimethylsilyl)borate | MAINLIB | 4325-85-3 | C9H27BO3Si3 | 278 | 93.19 | 909 | 918 |
| 7.35 | Trisiloxane, octamethyl- | MAINLIB | 107-51-7 | C8H24O2Si3 | 236 | 6.11 | 801 | 875 |
| 7.35 | 3,6-Dioxa-2,4,5,7-tetrasilaoctane, 2,2,4,4,5,5,7,7-octamethyl- | MAINLIB | 4342-25-0 | C10H30O2Si4 | 294 | 0.39 | 692 | 720 |

| Compound Structure | Delta | Hit Spectrum |
| --- | --- | --- |
|  |  |  |
|  |  |  |
|  |  |  |

| RT | Name | Library | Cas # | Molecular Formula | MolecularWeight | Probability | SI | RSI |
| --- | --- | --- | --- | --- | --- | --- | --- | --- |
| 8.53 | Cyclohexanone, 3,3,5-trimethyl- | MAINLIB | 873-94-9 | C9H16O | 140 | 71.57 | 812 | 858 |
| 8.53 | 2,4,6-Trimethyl-3-heptene | MAINLIB | NA | C10H20 | 140 | 11.76 | 748 | 842 |
| 8.53 | 3-Octene, 2,2-dimethyl- | MAINLIB | 86869-76-3 | C10H20 | 140 | 3.58 | 723 | 761 |

| Compound Structure | Delta | Hit Spectrum |
| --- | --- | --- |
|  |  |  |
|  |  |  |
|  |  |  |

| RT | Name | Library | Cas # | Molecular Formula | MolecularWeight | Probability | SI | RSI |
| --- | --- | --- | --- | --- | --- | --- | --- | --- |
| 8.99 | Propanoic acid, 2-[(trimethylsilyl)oxy]-, trimethylsilyl ester | MAINLIB | 17596-96-2 | C9H22O3Si2 | 234 | 90.27 | 941 | 962 |
| 8.99 | Silane, trimethyl[1-methyl-2-oxo-2-(trimethylsilyl)ethoxy]-, (R)- | MAINLIB | 55255-93-1 | C9H22O2Si2 | 218 | 6.37 | 838 | 845 |
| 8.99 | Butane, 2,3-bis(trimethylsiloxy)- | MAINLIB | 53274-85-4 | C10H26O2Si2 | 234 | 1.87 | 811 | 870 |

| Compound Structure | Delta | Hit Spectrum |
| --- | --- | --- |
|  |  |  |
|  |  |  |
|  |  |  |

| RT | Name | Library | Cas # | Molecular Formula | MolecularWeight | Probability | SI | RSI |
| --- | --- | --- | --- | --- | --- | --- | --- | --- |
| 9.30 | Acetic acid, [(trimethylsilyl)oxy]-, trimethylsilyl ester | MAINLIB | 33581-77-0 | C8H20O3Si2 | 220 | 81.31 | 901 | 935 |
| 9.30 | 3,7-Dioxa-2,8-disilanonane, 2,2,8,8-tetramethyl- | MAINLIB | 17887-80-8 | C9H24O2Si2 | 220 | 1.88 | 734 | 749 |
| 9.30 | Dimethyl(trimethylsilyl)ethoxysilane | MAINLIB | 18297-47-7 | C7H20OSi2 | 176 | 1.18 | 720 | 790 |

| Compound Structure | Delta | Hit Spectrum |
| --- | --- | --- |
|  |  |  |
|  |  |  |
|  |  |  |

| RT | Name | Library | Cas # | Molecular Formula | MolecularWeight | Probability | SI | RSI |
| --- | --- | --- | --- | --- | --- | --- | --- | --- |
| 9.89 | 1-(3-Methylbutyl)-2,3,4,6-tetramethylbenzene | MAINLIB | 116761-72-9 | C15H24 | 204 | 32.34 | 760 | 842 |
| 9.89 | Disiloxane, hexamethyl- | MAINLIB | 107-46-0 | C6H18OSi2 | 162 | 7.62 | 721 | 904 |
| 9.89 | Ethanedioic acid, bis(trimethylsilyl) ester | MAINLIB | 18294-04-7 | C8H18O4Si2 | 234 | 7.62 | 721 | 867 |

| Compound Structure | Delta | Hit Spectrum |
| --- | --- | --- |
|  |  |  |
|  |  |  |
|  |  |  |

| RT | Name | Library | Cas # | Molecular Formula | MolecularWeight | Probability | SI | RSI |
| --- | --- | --- | --- | --- | --- | --- | --- | --- |
| 12.95 | Urea, N,N'-bis(trimethylsilyl)- | MAINLIB | 18297-63-7 | C7H20N2OSi2 | 204 | 96.08 | 911 | 916 |
| 12.95 | tert-Butylpentamethyldisiloxane | MAINLIB | 67875-54-1 | C9H24OSi2 | 204 | 2.07 | 740 | 835 |
| 12.95 | Disiloxane, 1,3-bis(1,1-dimethylethyl)-1,1,3,3-tetramethyl- | MAINLIB | 67875-55-2 | C12H30OSi2 | 246 | 0.75 | 717 | 807 |

| Compound Structure | Delta | Hit Spectrum |
| --- | --- | --- |
|  |  |  |
|  |  |  |
|  |  |  |

| RT | Name | Library | Cas # | Molecular Formula | MolecularWeight | Probability | SI | RSI |
| --- | --- | --- | --- | --- | --- | --- | --- | --- |
| 13.02 | Benzoic acid trimethylsilyl ester | MAINLIB | 2078-12-8 | C10H14O2Si | 194 | 82.21 | 897 | 965 |
| 13.02 | Silanol, (1,1-dimethylethyl)dimethyl-, benzoate | MAINLIB | 75732-41-1 | C13H20O2Si | 236 | 16.10 | 843 | 882 |
| 13.02 | Methyl 3-hydroxy-3-phenylvalerate | MAINLIB | NA | C12H16O3 | 208 | 0.89 | 725 | 764 |

| Compound Structure | Delta | Hit Spectrum |
| --- | --- | --- |
|  |  |  |
|  |  |  |
|  |  |  |

| RT | Name | Library | Cas # | Molecular Formula | MolecularWeight | Probability | SI | RSI |
| --- | --- | --- | --- | --- | --- | --- | --- | --- |
| 13.07 | 3,6,9-Trioxa-2,10-disilaundecane, 2,2,10,10-tetramethyl- | MAINLIB | 16654-74-3 | C10H26O3Si2 | 250 | 71.86 | 836 | 898 |
| 13.07 | 3,6,9,12-Tetraoxa-2,13-disilatetradecane, 2,2,13,13-tetramethyl- | MAINLIB | 62185-58-4 | C12H30O4Si2 | 294 | 17.21 | 798 | 825 |
| 13.07 | 3,6,10,13-Tetraoxa-2,14-disilapentadecane, 2,2,14,14-tetramethyl- | MAINLIB | NA | C13H32O4Si2 | 308 | 4.12 | 760 | 791 |

| Compound Structure | Delta | Hit Spectrum |
| --- | --- | --- |
|  |  |  |
|  |  |  |
|  |  |  |

| RT | Name | Library | Cas # | Molecular Formula | MolecularWeight | Probability | SI | RSI |
| --- | --- | --- | --- | --- | --- | --- | --- | --- |
| 13.83 | Silanol, trimethyl-, phosphate (3:1) | MAINLIB | 10497-05-9 | C9H27O4PSi3 | 314 | 96.46 | 884 | 904 |
| 13.83 | 2-(4-Methoxyphenyl)-2-(4-trimethoxysilyloxy)propane | MAINLIB | NA | C19H26O2Si | 314 | 2.77 | 736 | 885 |
| 13.83 | [1,2,4]Oxadiazole, 5-(4-tert-butylphenoxymethyl)-3-(thiophen-2-yl)- | MAINLIB | NA | C17H18N2O2S | 314 | 0.21 | 639 | 803 |

| Compound Structure | Delta | Hit Spectrum |
| --- | --- | --- |
|  |  |  |
|  |  |  |
|  |  |  |

| RT | Name | Library | Cas # | Molecular Formula | MolecularWeight | Probability | SI | RSI |
| --- | --- | --- | --- | --- | --- | --- | --- | --- |
| 13.88 | Trimethylsilyl ether of glycerol | MAINLIB | 6787-10-6 | C12H32O3Si3 | 308 | 88.06 | 893 | 919 |
| 13.88 | Butane, 1,2,3-tris(trimethylsiloxy)- | MAINLIB | 33581-76-9 | C13H34O3Si3 | 322 | 4.22 | 770 | 796 |
| 13.88 | Silane, [(1-methoxy-1,3-propanediyl)bis(oxy)]bis[trimethyl- | MAINLIB | 62185-57-3 | C10H26O3Si2 | 250 | 4.06 | 769 | 828 |

| Compound Structure | Delta | Hit Spectrum |
| --- | --- | --- |
|  |  |  |
|  |  |  |
|  |  |  |

| RT | Name | Library | Cas # | Molecular Formula | MolecularWeight | Probability | SI | RSI |
| --- | --- | --- | --- | --- | --- | --- | --- | --- |
| 14.63 | Butanedioic acid, bis(trimethylsilyl) ester | MAINLIB | 40309-57-7 | C10H22O4Si2 | 262 | 83.71 | 937 | 969 |
| 14.63 | Propanedioic acid, methyl-, bis(trimethylsilyl) ester | MAINLIB | 40333-07-1 | C10H22O4Si2 | 262 | 12.18 | 867 | 888 |
| 14.63 | 5-Trimethylsilyloxy-n-valeric acid, trimethylsilyl ester | MAINLIB | NA | C11H26O3Si2 | 262 | 1.77 | 797 | 801 |

| Compound Structure | Delta | Hit Spectrum |
| --- | --- | --- |
|  |  |  |
|  |  |  |
|  |  |  |

| RT | Name | Library | Cas # | Molecular Formula | MolecularWeight | Probability | SI | RSI |
| --- | --- | --- | --- | --- | --- | --- | --- | --- |
| 15.14 | Propanoic acid, 2,3-bis[(trimethylsilyl)oxy]-, trimethylsilyl ester | MAINLIB | 38191-87-6 | C12H30O4Si3 | 322 | 94.73 | 926 | 927 |
| 15.14 | Tris(trimethylsiloxy)ethylene | MAINLIB | 69097-20-7 | C11H28O3Si3 | 292 | 0.94 | 716 | 728 |
| 15.14 | Ribonic acid, 2,3,4,5-tetrakis-O-(trimethylsilyl)-, trimethylsilyl ester | MAINLIB | 57197-35-0 | C20H50O6Si5 | 526 | 0.63 | 704 | 714 |

| Compound Structure | Delta | Hit Spectrum |
| --- | --- | --- |
|  |  |  |
|  |  |  |
|  |  |  |

| RT | Name | Library | Cas # | Molecular Formula | MolecularWeight | Probability | SI | RSI |
| --- | --- | --- | --- | --- | --- | --- | --- | --- |
| 15.31 | 2-Butenedioic acid (E)-, bis(trimethylsilyl) ester | MAINLIB | 17962-03-7 | C10H20O4Si2 | 260 | 90.53 | 934 | 959 |
| 15.31 | 2-Butenedioic acid (Z)-, bis(trimethylsilyl) ester | MAINLIB | 23508-82-9 | C10H20O4Si2 | 260 | 5.29 | 818 | 835 |
| 15.31 | 2-Pentenoic acid, 2-[(trimethylsilyl)oxy]-, trimethylsilyl ester | MAINLIB | 55045-17-5 | C11H24O3Si2 | 260 | 3.05 | 802 | 848 |

| Compound Structure | Delta | Hit Spectrum |
| --- | --- | --- |
|  |  |  |
|  |  |  |
|  |  |  |

| RT | Name | Library | Cas # | Molecular Formula | MolecularWeight | Probability | SI | RSI |
| --- | --- | --- | --- | --- | --- | --- | --- | --- |
| 15.50 | 5-Keto-2,2-dimethylheptanoic acid, ethyl(ester) | MAINLIB | NA | C11H20O3 | 200 | 8.48 | 626 | 643 |
| 15.50 | 3-Propionyloxytridecane | MAINLIB | NA | C16H32O2 | 256 | 6.32 | 618 | 685 |
| 15.50 | Butyl caprylate | MAINLIB | 589-75-3 | C12H24O2 | 200 | 4.84 | 611 | 622 |

| Compound Structure | Delta | Hit Spectrum |
| --- | --- | --- |
|  |  |  |
|  |  |  |
|  |  |  |

| RT | Name | Library | Cas # | Molecular Formula | MolecularWeight | Probability | SI | RSI |
| --- | --- | --- | --- | --- | --- | --- | --- | --- |
| 15.69 | Butane, 1,2,3-tris(trimethylsiloxy)- | MAINLIB | 33581-76-9 | C13H34O3Si3 | 322 | 20.10 | 693 | 730 |
| 15.69 | Sym-tetramethyl(diisopropyl)disiloxane | MAINLIB | 36957-90-1 | C10H26OSi2 | 218 | 16.98 | 689 | 734 |
| 15.69 | 2-Pentamethyldisilyloxypropane | MAINLIB | 78669-50-8 | C8H22OSi2 | 190 | 16.98 | 689 | 781 |

| Compound Structure | Delta | Hit Spectrum |
| --- | --- | --- |
|  |  |  |
|  |  |  |
|  |  |  |

| RT | Name | Library | Cas # | Molecular Formula | MolecularWeight | Probability | SI | RSI |
| --- | --- | --- | --- | --- | --- | --- | --- | --- |
| 16.49 | 1,3-Bis(trimethylsiloxy)benzene | MAINLIB | 4520-29-0 | C12H22O2Si2 | 254 | 73.64 | 736 | 803 |
| 16.49 | Silane, [1,4-phenylenebis(oxy)]bis[trimethyl- | MAINLIB | 2117-24-0 | C12H22O2Si2 | 254 | 13.07 | 676 | 872 |
| 16.49 | D-Ribonic acid, 5-deoxy-2,3-bis-O-(trimethylsilyl)-, ç-lactone | MAINLIB | 62338-19-6 | C11H24O4Si2 | 276 | 1.69 | 601 | 802 |

| Compound Structure | Delta | Hit Spectrum |
| --- | --- | --- |
|  |  |  |
|  |  |  |
|  |  |  |

| RT | Name | Library | Cas # | Molecular Formula | MolecularWeight | Probability | SI | RSI |
| --- | --- | --- | --- | --- | --- | --- | --- | --- |
| 17.26 | Malic acid, tris(trimethylsilyl) ester | MAINLIB | 65143-63-7 | C13H30O5Si3 | 350 | 17.51 | 735 | 797 |
| 17.26 | tert-Butylpentamethyldisiloxane | MAINLIB | 67875-54-1 | C9H24OSi2 | 204 | 10.08 | 719 | 831 |
| 17.26 | Malic acid, O-(trimethylsilyl)-, bis(trimethylsilyl)ester | MAINLIB | 107241-82-7 | C13H30O5Si3 | 350 | 5.50 | 702 | 716 |

| Compound Structure | Delta | Hit Spectrum |
| --- | --- | --- |
|  |  |  |
|  |  |  |
|  |  |  |

| RT | Name | Library | Cas # | Molecular Formula | MolecularWeight | Probability | SI | RSI |
| --- | --- | --- | --- | --- | --- | --- | --- | --- |
| 17.56 | Malic acid, tris(trimethylsilyl) ester | MAINLIB | 65143-63-7 | C13H30O5Si3 | 350 | 15.22 | 695 | 798 |
| 17.56 | Malic acid, O-(trimethylsilyl)-, bis(trimethylsilyl)ester | MAINLIB | 107241-82-7 | C13H30O5Si3 | 350 | 10.13 | 683 | 727 |
| 17.56 | Pentanoic acid, 3-methyl-3,5-bis[(trimethylsilyl)oxy]-, methyl ester | MAINLIB | 56051-93-5 | C13H30O4Si2 | 306 | 6.34 | 669 | 705 |

| Compound Structure | Delta | Hit Spectrum |
| --- | --- | --- |
|  |  |  |
|  |  |  |
|  |  |  |

| RT | Name | Library | Cas # | Molecular Formula | MolecularWeight | Probability | SI | RSI |
| --- | --- | --- | --- | --- | --- | --- | --- | --- |
| 18.48 | Malic acid, tris(trimethylsilyl) ester | MAINLIB | 65143-63-7 | C13H30O5Si3 | 350 | 45.67 | 905 | 923 |
| 18.48 | Malic acid, O-(trimethylsilyl)-, bis(trimethylsilyl)ester | MAINLIB | 107241-82-7 | C13H30O5Si3 | 350 | 42.12 | 903 | 903 |
| 18.48 | Butanedioic acid, [(trimethylsilyl)oxy]-, bis(trimethylsilyl) ester | MAINLIB | 38166-11-9 | C13H30O5Si3 | 350 | 11.02 | 870 | 890 |

| Compound Structure | Delta | Hit Spectrum |
| --- | --- | --- |
|  |  |  |
|  |  |  |
|  |  |  |

| RT | Name | Library | Cas # | Molecular Formula | MolecularWeight | Probability | SI | RSI |
| --- | --- | --- | --- | --- | --- | --- | --- | --- |
| 18.77 | Benzoic acid, 2-[(trimethylsilyl)oxy]-, trimethylsilyl ester | MAINLIB | 3789-85-3 | C13H22O3Si2 | 282 | 88.05 | 827 | 899 |
| 18.77 | p-Trimethylsilyloxyphenyl-(trimethylsilyloxy)trimethylsilylacrylate | MAINLIB | NA | C18H34O4Si3 | 398 | 2.57 | 680 | 805 |
| 18.77 | Silane, [[3,3-dimethyl-4-methylene-2-(trimethylsilyl)-1-cyclopenten-1-yl]methoxy]trimethyl- | MAINLIB | 95798-07-5 | C15H30OSi2 | 282 | 1.09 | 659 | 691 |

| Compound Structure | Delta | Hit Spectrum |
| --- | --- | --- |
|  |  |  |
|  |  |  |
|  |  |  |

| RT | Name | Library | Cas # | Molecular Formula | MolecularWeight | Probability | SI | RSI |
| --- | --- | --- | --- | --- | --- | --- | --- | --- |
| 19.03 | L-Proline, 5-oxo-1-(trimethylsilyl)-, trimethylsilyl ester | MAINLIB | 30274-77-2 | C11H23NO3Si2 | 273 | 80.27 | 913 | 924 |
| 19.03 | N,O-Bis-(trimethylsilyl)-2-pyrrolidone carboxylic acid | MAINLIB | 213608-51-6 | C11H23NO3Si2 | 273 | 12.42 | 846 | 917 |
| 19.03 | 2-Piperidinecarboxylic acid, 1-(trimethylsilyl)-, trimethylsilyl ester | MAINLIB | 55255-44-2 | C12H27NO2Si2 | 273 | 3.78 | 821 | 827 |

| Compound Structure | Delta | Hit Spectrum |
| --- | --- | --- |
|  |  |  |
|  |  |  |
|  |  |  |

| RT | Name | Library | Cas # | Molecular Formula | MolecularWeight | Probability | SI | RSI |
| --- | --- | --- | --- | --- | --- | --- | --- | --- |
| 19.31 | 2-Oxiranemethanol, à,alpha.-dimethyl-3-[1-(t-butyldimethylsilyloxy)pentyl]- | MAINLIB | NA | C16H34O3Si | 302 | 29.14 | 653 | 664 |
| 19.31 | 2-Oxiranemethanol, à-(1-methylethyl)-3-[1-(trimethylsilyloxy)pentyl]- | MAINLIB | NA | C14H30O3Si | 274 | 21.74 | 645 | 692 |
| 19.31 | 7-Oxooctanoic acid, 2-trimethylsilylethyl ester | MAINLIB | 65690-30-4 | C13H26O3Si | 258 | 12.52 | 629 | 648 |

| Compound Structure | Delta | Hit Spectrum |
| --- | --- | --- |
|  |  |  |
|  |  |  |
|  |  |  |

| RT | Name | Library | Cas # | Molecular Formula | MolecularWeight | Probability | SI | RSI |
| --- | --- | --- | --- | --- | --- | --- | --- | --- |
| 20.26 | Erythro-Pentonic acid, 2-deoxy-3,4,5-tris-O-(trimethylsilyl)-, trimethylsilyl ester | MAINLIB | 74742-30-6 | C17H42O5Si4 | 438 | 17.43 | 669 | 694 |
| 20.26 | d-Erythrotetrofuranose, tris-O-(trimethylsilyl)- | MAINLIB | NA | C13H32O4Si3 | 336 | 14.05 | 664 | 760 |
| 20.26 | Malic acid, tris(trimethylsilyl) ester | MAINLIB | 65143-63-7 | C13H30O5Si3 | 350 | 5.13 | 641 | 804 |

| Compound Structure | Delta | Hit Spectrum |
| --- | --- | --- |
|  |  |  |
|  |  |  |
|  |  |  |

| RT | Name | Library | Cas # | Molecular Formula | MolecularWeight | Probability | SI | RSI |
| --- | --- | --- | --- | --- | --- | --- | --- | --- |
| 20.68 | Pentanedioic acid, 3-methyl-3-[(trimethylsilyl)oxy]-, bis(trimethylsilyl) ester | MAINLIB | 55590-95-9 | C15H34O5Si3 | 378 | 92.80 | 883 | 906 |
| 20.68 | Hexanedioic acid, 3-trimethylsiloxy-, bis(trimethylsilyl) ester | MAINLIB | 73105-00-7 | C15H34O5Si3 | 378 | 4.04 | 756 | 771 |
| 20.68 | Acetic acid, [(tert-butyldimethylsilyl)oxy]-, tert-butyldimethylsilyl ester | MAINLIB | 67226-76-0 | C14H32O3Si2 | 304 | 0.35 | 668 | 798 |

| Compound Structure | Delta | Hit Spectrum |
| --- | --- | --- |
|  |  |  |
|  |  |  |
|  |  |  |

| RT | Name | Library | Cas # | Molecular Formula | MolecularWeight | Probability | SI | RSI |
| --- | --- | --- | --- | --- | --- | --- | --- | --- |
| 20.99 | Benzoic acid, 4-[(trimethylsilyl)oxy]-, trimethylsilyl ester | MAINLIB | 2078-13-9 | C13H22O3Si2 | 282 | 74.21 | 863 | 919 |
| 20.99 | Benzoic acid, 3-[(trimethylsilyl)oxy]-, trimethylsilyl ester | MAINLIB | 3782-84-1 | C13H22O3Si2 | 282 | 24.87 | 839 | 883 |
| 20.99 | Urocanic acid, N,O-bis(trimethylsilyl)- | MAINLIB | NA | C12H22N2O2Si2 | 282 | 0.36 | 647 | 672 |

| Compound Structure | Delta | Hit Spectrum |
| --- | --- | --- |
|  |  |  |
|  |  |  |
|  |  |  |

| RT | Name | Library | Cas # | Molecular Formula | MolecularWeight | Probability | SI | RSI |
| --- | --- | --- | --- | --- | --- | --- | --- | --- |
| 21.12 | 2-Piperidinecarboxylic acid, 1-(trimethylsilyl)-, trimethylsilyl ester | MAINLIB | 55255-44-2 | C12H27NO2Si2 | 273 | 31.23 | 645 | 697 |
| 21.12 | Cyclopentanecarboxylic acid, 1-amino-, bis(trimethylsilyl) deriv. | MAINLIB | 56273-05-3 | C12H27NO2Si2 | 273 | 20.18 | 632 | 855 |
| 21.12 | L-Proline, 5-oxo-1-(trimethylsilyl)-, trimethylsilyl ester | MAINLIB | 30274-77-2 | C11H23NO3Si2 | 273 | 18.61 | 630 | 858 |

| Compound Structure | Delta | Hit Spectrum |
| --- | --- | --- |
|  |  |  |
|  |  |  |
|  |  |  |

| RT | Name | Library | Cas # | Molecular Formula | MolecularWeight | Probability | SI | RSI |
| --- | --- | --- | --- | --- | --- | --- | --- | --- |
| 21.33 | 2,3,4,5-Tetrahydroxypentanoic acid-1,4-lactone, tris(trimethylsilyl)- | MAINLIB | NA | C14H32O5Si3 | 364 | 76.13 | 881 | 928 |
| 21.33 | D-Arabinonic acid, 2,3,5-tris-O-(trimethylsilyl)-, ç-lactone | MAINLIB | 32384-55-7 | C14H32O5Si3 | 364 | 13.01 | 819 | 888 |
| 21.33 | Xylonic acid, 2,3,5-tris-O-(trimethylsilyl)-, ç-lactone, D- | MAINLIB | 10589-36-3 | C14H32O5Si3 | 364 | 6.71 | 801 | 870 |

| Compound Structure | Delta | Hit Spectrum |
| --- | --- | --- |
|  |  |  |
|  |  |  |
|  |  |  |

| RT | Name | Library | Cas # | Molecular Formula | MolecularWeight | Probability | SI | RSI |
| --- | --- | --- | --- | --- | --- | --- | --- | --- |
| 22.61 | à-D-Galactopyranoside, methyl 2,6-bis-O-(trimethylsilyl)-, cyclic methylboronate | MAINLIB | 54400-89-4 | C14H31BO6Si2 | 362 | 9.02 | 698 | 717 |
| 22.61 | à-D-Galactopyranose, 1,2,3-tris-O-(trimethylsilyl)-, cyclic methylboronate | MAINLIB | 56196-95-3 | C16H37BO6Si3 | 420 | 9.02 | 698 | 717 |
| 22.61 | 2,3,4,5-Tetrahydroxypentanoic acid-1,4-lactone, tris(trimethylsilyl)- | MAINLIB | NA | C14H32O5Si3 | 364 | 7.62 | 694 | 820 |

| Compound Structure | Delta | Hit Spectrum |
| --- | --- | --- |
|  |  |  |
|  |  |  |
|  |  |  |

| RT | Name | Library | Cas # | Molecular Formula | MolecularWeight | Probability | SI | RSI |
| --- | --- | --- | --- | --- | --- | --- | --- | --- |
| 23.83 | Phosphoric acid, bis(trimethylsilyl) 2,3-bis[(trimethylsilyl)oxy]propyl ester | MAINLIB | 31038-11-6 | C15H41O6PSi4 | 460 | 61.13 | 800 | 886 |
| 23.83 | Phosphoric acid, 4-oxo-2,3-bis[(trimethylsilyl)oxy]butyl bis(trimethylsilyl) ester, [R-(R*,R*)]- | MAINLIB | 55723-94-9 | C16H41O7PSi4 | 488 | 11.05 | 741 | 788 |
| 23.83 | D-erythro-2-Pentulose, 1,3,4-tris-O-(trimethylsilyl)-, 5-[bis(trimethylsilyl) phosphate] | MAINLIB | 55520-87-1 | C20H51O8PSi5 | 590 | 7.58 | 730 | 766 |

| Compound Structure | Delta | Hit Spectrum |
| --- | --- | --- |
|  |  |  |
|  |  |  |
|  |  |  |

| RT | Name | Library | Cas # | Molecular Formula | MolecularWeight | Probability | SI | RSI |
| --- | --- | --- | --- | --- | --- | --- | --- | --- |
| 23.98 | à-D-Glucopyranosiduronic acid, 3-(5-ethylhexahydro-2,4,6-trioxo-5-pyrimidinyl)-1,1-dimethylpropyl 2,3,4-tris-O-(trimethylsilyl)-, methyl ester | MAINLIB | 55556-81-5 | C27H52N2O10Si3 | 648 | 9.82 | 637 | 638 |
| 23.98 | á-D-Galactopyranoside, methyl 2,6-bis-O-(trimethylsilyl)-, cyclic butylboronate | MAINLIB | 56211-13-3 | C17H37BO6Si2 | 404 | 7.71 | 631 | 668 |
| 23.98 | tert-Hexadecanethiol | MAINLIB | 25360-09-2 | C16H34S | 258 | 6.06 | 625 | 675 |

| Compound Structure | Delta | Hit Spectrum |
| --- | --- | --- |
|  |  |  |
|  |  |  |
|  |  |  |

| RT | Name | Library | Cas # | Molecular Formula | MolecularWeight | Probability | SI | RSI |
| --- | --- | --- | --- | --- | --- | --- | --- | --- |
| 24.48 | Citric acid, ethyl ester, tri-TMS | MAINLIB | NA | C17H36O7Si3 | 436 | 64.49 | 736 | 775 |
| 24.48 | 1,2,3-Propanetricarboxylic acid, 2-[(trimethylsilyl)oxy]-, tris(trimethylsilyl) ester | MAINLIB | 14330-97-3 | C18H40O7Si4 | 480 | 5.63 | 648 | 788 |
| 24.48 | Monononanoin, bis(trimethylsilyl)- deriv. | MAINLIB | NA | C18H40O4Si2 | 376 | 5.41 | 647 | 695 |

| Compound Structure | Delta | Hit Spectrum |
| --- | --- | --- |
|  |  |  |
|  |  |  |
|  |  |  |

| RT | Name | Library | Cas # | Molecular Formula | MolecularWeight | Probability | SI | RSI |
| --- | --- | --- | --- | --- | --- | --- | --- | --- |
| 24.59 | Benzoic acid, 3,4-bis[(trimethylsilyl)oxy]-, trimethylsilyl ester | MAINLIB | 2347-40-2 | C16H30O4Si3 | 370 | 96.47 | 832 | 921 |
| 24.59 | 3,5-Bis(trimethylsiloxy)benzoic acid, trimethylsilyl ester | MAINLIB | 79314-27-5 | C16H30O4Si3 | 370 | 1.77 | 653 | 677 |
| 24.59 | 2-Phenyl-1,2-bis(trimethylsilyloxy)propane | MAINLIB | 294847-15-7 | C15H28O2Si2 | 296 | 0.32 | 594 | 918 |

| Compound Structure | Delta | Hit Spectrum |
| --- | --- | --- |
|  |  |  |
|  |  |  |
|  |  |  |

| RT | Name | Library | Cas # | Molecular Formula | MolecularWeight | Probability | SI | RSI |
| --- | --- | --- | --- | --- | --- | --- | --- | --- |
| 24.68 | D-Ribofuranose, 1,2,3,5-tetrakis-O-(trimethylsilyl)- | MAINLIB | 56271-69-3 | C17H42O5Si4 | 438 | 15.92 | 763 | 835 |
| 24.68 | D-Xylofuranose, 1,2,3,5-tetrakis-O-(trimethylsilyl)- | MAINLIB | 56271-68-2 | C17H42O5Si4 | 438 | 12.20 | 756 | 820 |
| 24.68 | D-Fructose, 1,3,4,5,6-pentakis-O-(trimethylsilyl)- | MAINLIB | 19126-98-8 | C21H52O6Si5 | 540 | 10.78 | 753 | 765 |

| Compound Structure | Delta | Hit Spectrum |
| --- | --- | --- |
|  |  |  |
|  |  |  |
|  |  |  |

| RT | Name | Library | Cas # | Molecular Formula | MolecularWeight | Probability | SI | RSI |
| --- | --- | --- | --- | --- | --- | --- | --- | --- |
| 24.82 | 2-Keto-d-gluconic acid, pentakis(O-trimethylsilyl)- | MAINLIB | NA | C21H50O7Si5 | 554 | 18.31 | 766 | 804 |
| 24.82 | D-Fructose, 1,3,4,5,6-pentakis-O-(trimethylsilyl)- | MAINLIB | 19126-98-8 | C21H52O6Si5 | 540 | 15.46 | 762 | 783 |
| 24.82 | D-Xylofuranose, 1,2,3,5-tetrakis-O-(trimethylsilyl)- | MAINLIB | 56271-68-2 | C17H42O5Si4 | 438 | 9.99 | 749 | 832 |

| Compound Structure | Delta | Hit Spectrum |
| --- | --- | --- |
|  |  |  |
|  |  |  |
|  |  |  |

| RT | Name | Library | Cas # | Molecular Formula | MolecularWeight | Probability | SI | RSI |
| --- | --- | --- | --- | --- | --- | --- | --- | --- |
| 24.90 | D-Fructose, 1,3,4,5,6-pentakis-O-(trimethylsilyl)- | MAINLIB | 19126-98-8 | C21H52O6Si5 | 540 | 46.73 | 866 | 886 |
| 24.90 | Sorbopyranose, 1,2,3,4,5-pentakis-O-(trimethylsilyl)-, L- | MAINLIB | 30645-02-4 | C21H52O6Si5 | 540 | 22.69 | 847 | 880 |
| 24.90 | D-Xylopyranose, 1,2,3,4-tetrakis-O-(trimethylsilyl)- | MAINLIB | 55555-45-8 | C17H42O5Si4 | 438 | 1.98 | 759 | 858 |

| Compound Structure | Delta | Hit Spectrum |
| --- | --- | --- |
|  |  |  |
|  |  |  |
|  |  |  |

| RT | Name | Library | Cas # | Molecular Formula | MolecularWeight | Probability | SI | RSI |
| --- | --- | --- | --- | --- | --- | --- | --- | --- |
| 25.01 | á-DL-Arabinopyranose, 1,2,3,4-tetrakis-O-(trimethylsilyl)- | MAINLIB | 56271-64-8 | C17H42O5Si4 | 438 | 13.09 | 728 | 812 |
| 25.01 | á-DL-Lyxopyranose, 1,2,3,4-tetrakis-O-(trimethylsilyl)- | MAINLIB | 56271-66-0 | C17H42O5Si4 | 438 | 11.56 | 725 | 801 |
| 25.01 | Erythro-Pentonic acid, 2-deoxy-3,4,5-tris-O-(trimethylsilyl)-, trimethylsilyl ester | MAINLIB | 74742-30-6 | C17H42O5Si4 | 438 | 8.86 | 718 | 797 |

| Compound Structure | Delta | Hit Spectrum |
| --- | --- | --- |
|  |  |  |
|  |  |  |
|  |  |  |

| RT | Name | Library | Cas # | Molecular Formula | MolecularWeight | Probability | SI | RSI |
| --- | --- | --- | --- | --- | --- | --- | --- | --- |
| 25.21 | á-DL-Lyxopyranose, 1,2,3,4-tetrakis-O-(trimethylsilyl)- | MAINLIB | 56271-66-0 | C17H42O5Si4 | 438 | 12.79 | 732 | 794 |
| 25.21 | á-DL-Arabinopyranose, 1,2,3,4-tetrakis-O-(trimethylsilyl)- | MAINLIB | 56271-64-8 | C17H42O5Si4 | 438 | 10.80 | 728 | 792 |
| 25.21 | à-D-Mannopyranoside, methyl 2,3-bis-O-(trimethylsilyl)-, cyclic butylboronate | MAINLIB | 56211-09-7 | C17H37BO6Si2 | 404 | 6.54 | 713 | 766 |

| Compound Structure | Delta | Hit Spectrum |
| --- | --- | --- |
|  |  |  |
|  |  |  |
|  |  |  |

| RT | Name | Library | Cas # | Molecular Formula | MolecularWeight | Probability | SI | RSI |
| --- | --- | --- | --- | --- | --- | --- | --- | --- |
| 25.35 | á-DL-Arabinopyranose, 1,2,3,4-tetrakis-O-(trimethylsilyl)- | MAINLIB | 56271-64-8 | C17H42O5Si4 | 438 | 16.35 | 813 | 858 |
| 25.35 | D-Ribofuranose, 1,2,3,5-tetrakis-O-(trimethylsilyl)- | MAINLIB | 56271-69-3 | C17H42O5Si4 | 438 | 13.81 | 809 | 879 |
| 25.35 | D-Xylofuranose, 1,2,3,5-tetrakis-O-(trimethylsilyl)- | MAINLIB | 56271-68-2 | C17H42O5Si4 | 438 | 13.28 | 808 | 857 |

| Compound Structure | Delta | Hit Spectrum |
| --- | --- | --- |
|  |  |  |
|  |  |  |
|  |  |  |

| RT | Name | Library | Cas # | Molecular Formula | MolecularWeight | Probability | SI | RSI |
| --- | --- | --- | --- | --- | --- | --- | --- | --- |
| 25.45 | D-Fructose, 1,3,4,5,6-pentakis-O-(trimethylsilyl)- | MAINLIB | 19126-98-8 | C21H52O6Si5 | 540 | 33.15 | 797 | 873 |
| 25.45 | á-DL-Lyxopyranose, 1,2,3,4-tetrakis-O-(trimethylsilyl)- | MAINLIB | 56271-66-0 | C17H42O5Si4 | 438 | 8.85 | 765 | 839 |
| 25.45 | á-DL-Arabinopyranose, 1,2,3,4-tetrakis-O-(trimethylsilyl)- | MAINLIB | 56271-64-8 | C17H42O5Si4 | 438 | 5.36 | 750 | 829 |

| Compound Structure | Delta | Hit Spectrum |
| --- | --- | --- |
|  |  |  |
|  |  |  |
|  |  |  |

| RT | Name | Library | Cas # | Molecular Formula | MolecularWeight | Probability | SI | RSI |
| --- | --- | --- | --- | --- | --- | --- | --- | --- |
| 25.61 | á-DL-Lyxopyranose, 1,2,3,4-tetrakis-O-(trimethylsilyl)- | MAINLIB | 56271-66-0 | C17H42O5Si4 | 438 | 9.19 | 742 | 844 |
| 25.61 | D-Ribopyranose, 1,2,3,4-tetrakis-O-(trimethylsilyl)- | MAINLIB | 56271-70-6 | C17H42O5Si4 | 438 | 7.04 | 735 | 836 |
| 25.61 | á-D-Xylopyranose, 1,2,3,4-tetrakis-O-(trimethylsilyl)- | MAINLIB | 18623-27-3 | C17H42O5Si4 | 438 | 6.49 | 733 | 850 |

| Compound Structure | Delta | Hit Spectrum |
| --- | --- | --- |
|  |  |  |
|  |  |  |
|  |  |  |

| RT | Name | Library | Cas # | Molecular Formula | MolecularWeight | Probability | SI | RSI |
| --- | --- | --- | --- | --- | --- | --- | --- | --- |
| 25.68 | à-D-Mannopyranoside, methyl 2,3-bis-O-(trimethylsilyl)-, cyclic butylboronate | MAINLIB | 56211-09-7 | C17H37BO6Si2 | 404 | 8.64 | 646 | 685 |
| 25.68 | á-DL-Arabinopyranose, 1,2,3,4-tetrakis-O-(trimethylsilyl)- | MAINLIB | 56271-64-8 | C17H42O5Si4 | 438 | 5.23 | 631 | 687 |
| 25.68 | á-L-Galactopyranose, 6-deoxy-1,2,3,4-tetrakis-O-(trimethylsilyl)- | MAINLIB | 32727-31-4 | C18H44O5Si4 | 452 | 4.62 | 628 | 700 |

| Compound Structure | Delta | Hit Spectrum |
| --- | --- | --- |
|  |  |  |
|  |  |  |
|  |  |  |

| RT | Name | Library | Cas # | Molecular Formula | MolecularWeight | Probability | SI | RSI |
| --- | --- | --- | --- | --- | --- | --- | --- | --- |
| 26.15 | Glucopyranose, pentakis-O-trimethylsilyl- | MAINLIB | NA | C21H52O6Si5 | 540 | 12.80 | 913 | 914 |
| 26.15 | D-Glucose, 2,3,4,5,6-pentakis-O-(trimethylsilyl)- | MAINLIB | 6736-97-6 | C21H52O6Si5 | 540 | 10.06 | 907 | 964 |
| 26.15 | Glucopyranose, 1,2,3,4,6-pentakis-O-(trimethylsilyl)-, D- | MAINLIB | 19126-99-9 | C21H52O6Si5 | 540 | 10.06 | 907 | 914 |

| Compound Structure | Delta | Hit Spectrum |
| --- | --- | --- |
|  |  |  |
|  |  |  |
|  |  |  |

| RT | Name | Library | Cas # | Molecular Formula | MolecularWeight | Probability | SI | RSI |
| --- | --- | --- | --- | --- | --- | --- | --- | --- |
| 26.34 | Glucopyranose, pentakis-O-trimethylsilyl- | MAINLIB | NA | C21H52O6Si5 | 540 | 11.42 | 864 | 879 |
| 26.34 | Glucopyranose, 1,2,3,4,6-pentakis-O-(trimethylsilyl)-, D- | MAINLIB | 19126-99-9 | C21H52O6Si5 | 540 | 10.09 | 861 | 909 |
| 26.34 | D-Xylose, tetrakis(trimethylsilyl)- | MAINLIB | 18623-22-8 | C17H42O5Si4 | 438 | 8.52 | 857 | 926 |

| Compound Structure | Delta | Hit Spectrum |
| --- | --- | --- |
|  |  |  |
|  |  |  |
|  |  |  |

| RT | Name | Library | Cas # | Molecular Formula | MolecularWeight | Probability | SI | RSI |
| --- | --- | --- | --- | --- | --- | --- | --- | --- |
| 26.48 | Cinnamic acid, p-(trimethylsiloxy)-, trimethylsilyl ester | MAINLIB | 10517-30-3 | C15H24O3Si2 | 308 | 90.41 | 818 | 936 |
| 26.48 | Cinnamic acid, m-(trimethylsiloxy)-, trimethylsilyl ester | MAINLIB | 32342-01-1 | C15H24O3Si2 | 308 | 7.64 | 727 | 874 |
| 26.48 | Cinnamic acid, o-(trimethylsiloxy)-, trimethylsilyl ester | MAINLIB | 32426-62-3 | C15H24O3Si2 | 308 | 0.57 | 628 | 811 |

| Compound Structure | Delta | Hit Spectrum |
| --- | --- | --- |
|  |  |  |
|  |  |  |
|  |  |  |

| RT | Name | Library | Cas # | Molecular Formula | MolecularWeight | Probability | SI | RSI |
| --- | --- | --- | --- | --- | --- | --- | --- | --- |
| 26.57 | Acrylic acid, 2,3-bis[(trimethylsilyl)oxy]-, trimethylsilyl ester | MAINLIB | NA | C12H28O4Si3 | 320 | 40.73 | 714 | 757 |
| 26.57 | O,O,O'-Tris-trimethylsilylmalonate | MAINLIB | 40333-06-0 | C12H28O4Si3 | 320 | 39.15 | 713 | 761 |
| 26.57 | Inosose-2, 1,3,4,5,6-pentakis-O-(trimethylsilyl)-, myo- | MAINLIB | 14251-19-5 | C21H50O6Si5 | 538 | 6.69 | 651 | 671 |

| Compound Structure | Delta | Hit Spectrum |
| --- | --- | --- |
|  |  |  |
|  |  |  |
|  |  |  |

| RT | Name | Library | Cas # | Molecular Formula | MolecularWeight | Probability | SI | RSI |
| --- | --- | --- | --- | --- | --- | --- | --- | --- |
| 27.22 | Hexadecanoic acid, ethyl ester | MAINLIB | 628-97-7 | C18H36O2 | 284 | 68.70 | 857 | 888 |
| 27.22 | Eicosanoic acid, ethyl ester | MAINLIB | 18281-05-5 | C22H44O2 | 340 | 10.63 | 790 | 844 |
| 27.22 | Pentadecanoic acid, ethyl ester | MAINLIB | 41114-00-5 | C17H34O2 | 270 | 8.35 | 784 | 850 |

| Compound Structure | Delta | Hit Spectrum |
| --- | --- | --- |
|  |  |  |
|  |  |  |
|  |  |  |

| RT | Name | Library | Cas # | Molecular Formula | MolecularWeight | Probability | SI | RSI |
| --- | --- | --- | --- | --- | --- | --- | --- | --- |
| 27.35 | D-Xylofuranose, 1,2,3,5-tetrakis-O-(trimethylsilyl)- | MAINLIB | 56271-68-2 | C17H42O5Si4 | 438 | 20.68 | 781 | 828 |
| 27.35 | D-Ribofuranose, 1,2,3,5-tetrakis-O-(trimethylsilyl)- | MAINLIB | 56271-69-3 | C17H42O5Si4 | 438 | 11.91 | 765 | 842 |
| 27.35 | á-DL-Arabinopyranose, 1,2,3,4-tetrakis-O-(trimethylsilyl)- | MAINLIB | 56271-64-8 | C17H42O5Si4 | 438 | 9.12 | 758 | 815 |

| Compound Structure | Delta | Hit Spectrum |
| --- | --- | --- |
|  |  |  |
|  |  |  |
|  |  |  |

| RT | Name | Library | Cas # | Molecular Formula | MolecularWeight | Probability | SI | RSI |
| --- | --- | --- | --- | --- | --- | --- | --- | --- |
| 27.65 | Glucopyranose, 1,2,3,4,6-pentakis-O-(trimethylsilyl)-, D- | MAINLIB | 19126-99-9 | C21H52O6Si5 | 540 | 15.59 | 922 | 929 |
| 27.65 | Glucopyranose, pentakis-O-trimethylsilyl- | MAINLIB | NA | C21H52O6Si5 | 540 | 13.78 | 919 | 921 |
| 27.65 | Talose, 2,3,4,5,6-pentakis-O-(trimethylsilyl)- | MAINLIB | 56192-85-9 | C21H52O6Si5 | 540 | 9.73 | 909 | 924 |

| Compound Structure | Delta | Hit Spectrum |
| --- | --- | --- |
|  |  |  |
|  |  |  |
|  |  |  |

| RT | Name | Library | Cas # | Molecular Formula | MolecularWeight | Probability | SI | RSI |
| --- | --- | --- | --- | --- | --- | --- | --- | --- |
| 27.77 | à-D-Galactopyranose, 1,2,3-tris-O-(trimethylsilyl)-, cyclic methylboronate | MAINLIB | 56196-95-3 | C16H37BO6Si3 | 420 | 14.71 | 715 | 748 |
| 27.77 | á-D-Galactopyranoside, methyl 2,6-bis-O-(trimethylsilyl)-, cyclic methylboronate | MAINLIB | 56211-06-4 | C14H31BO6Si2 | 362 | 11.86 | 710 | 743 |
| 27.77 | D-Glucose, 6-O-à-D-galactopyranosyl-, bis-O-(trimethylsilyl) deriv., cyclic tris(methylboronate) | MAINLIB | 72347-76-3 | C21H41B3O11Si2 | 558 | 11.40 | 709 | 741 |

| Compound Structure | Delta | Hit Spectrum |
| --- | --- | --- |
|  |  |  |
|  |  |  |
|  |  |  |

| RT | Name | Library | Cas # | Molecular Formula | MolecularWeight | Probability | SI | RSI |
| --- | --- | --- | --- | --- | --- | --- | --- | --- |
| 28.13 | Hexadecanoic acid, trimethylsilyl ester | MAINLIB | 55520-89-3 | C19H40O2Si | 328 | 93.20 | 878 | 889 |
| 28.13 | Tetradecanoic acid, trimethylsilyl ester | MAINLIB | 18603-17-3 | C17H36O2Si | 300 | 2.23 | 713 | 839 |
| 28.13 | Octadecanoic acid, trimethylsilyl ester | MAINLIB | 18748-91-9 | C21H44O2Si | 356 | 1.97 | 710 | 772 |

| Compound Structure | Delta | Hit Spectrum |
| --- | --- | --- |
|  |  |  |
|  |  |  |
|  |  |  |

| RT | Name | Library | Cas # | Molecular Formula | MolecularWeight | Probability | SI | RSI |
| --- | --- | --- | --- | --- | --- | --- | --- | --- |
| 28.85 | Tetradecane, 2,6,10-trimethyl- | MAINLIB | 14905-56-7 | C17H36 | 240 | 6.25 | 804 | 846 |
| 28.85 | Tetratetracontane | MAINLIB | 7098-22-8 | C44H90 | 618 | 5.28 | 800 | 831 |
| 28.85 | Heptadecane, 2,6,10,15-tetramethyl- | MAINLIB | 54833-48-6 | C21H44 | 296 | 4.25 | 795 | 870 |

| Compound Structure | Delta | Hit Spectrum |
| --- | --- | --- |
|  |  |  |
|  |  |  |
|  |  |  |

| RT | Name | Library | Cas # | Molecular Formula | MolecularWeight | Probability | SI | RSI |
| --- | --- | --- | --- | --- | --- | --- | --- | --- |
| 29.21 | à-D-Glucopyranoside, methyl 2-(acetylamino)-2-deoxy-3-O-(trimethylsilyl)-, cyclic methylboronate | MAINLIB | 54477-01-9 | C13H26BNO6Si | 331 | 33.48 | 739 | 775 |
| 29.21 | á-D-Galactopyranoside, methyl 2,3-bis-O-(trimethylsilyl)-, cyclic methylboronate | MAINLIB | 56211-08-6 | C14H31BO6Si2 | 362 | 8.94 | 707 | 735 |
| 29.21 | á-D-Galactopyranoside, methyl 2,6-bis-O-(trimethylsilyl)-, cyclic methylboronate | MAINLIB | 56211-06-4 | C14H31BO6Si2 | 362 | 7.90 | 704 | 736 |

| Compound Structure | Delta | Hit Spectrum |
| --- | --- | --- |
|  |  |  |
|  |  |  |
|  |  |  |

| RT | Name | Library | Cas # | Molecular Formula | MolecularWeight | Probability | SI | RSI |
| --- | --- | --- | --- | --- | --- | --- | --- | --- |
| 29.30 | Myo-Inositol, 1,2,3,4,5,6-hexakis-O-(trimethylsilyl)- | MAINLIB | 2582-79-8 | C24H60O6Si6 | 612 | 82.56 | 910 | 927 |
| 29.30 | Inositol, 1,2,3,4,5,6-hexakis-O-(trimethylsilyl)-, epi- | MAINLIB | 29267-01-4 | C24H60O6Si6 | 612 | 2.06 | 750 | 844 |
| 29.30 | Inositol, 1,2,3,4,5,6-hexakis-O-(trimethylsilyl)-, cis- | MAINLIB | 29412-27-9 | C24H60O6Si6 | 612 | 1.82 | 747 | 827 |

| Compound Structure | Delta | Hit Spectrum |
| --- | --- | --- |
|  |  |  |
|  |  |  |
|  |  |  |

| RT | Name | Library | Cas # | Molecular Formula | MolecularWeight | Probability | SI | RSI |
| --- | --- | --- | --- | --- | --- | --- | --- | --- |
| 29.57 | Heptadecanoic acid, trimethylsilyl ester | MAINLIB | 55517-58-3 | C20H42O2Si | 342 | 58.86 | 782 | 883 |
| 29.57 | Tetradecanoic acid, trimethylsilyl ester | MAINLIB | 18603-17-3 | C17H36O2Si | 300 | 3.72 | 671 | 763 |
| 29.57 | Undecanoic acid, 11-chloro-, trimethylsilyl ester | MAINLIB | 26305-98-6 | C14H29ClO2Si | 292 | 2.62 | 661 | 823 |

| Compound Structure | Delta | Hit Spectrum |
| --- | --- | --- |
|  |  |  |
|  |  |  |
|  |  |  |

| RT | Name | Library | Cas # | Molecular Formula | MolecularWeight | Probability | SI | RSI |
| --- | --- | --- | --- | --- | --- | --- | --- | --- |
| 29.81 | 9,12-Octadecadienoic acid, ethyl ester | MAINLIB | 7619-08-1 | C20H36O2 | 308 | 25.12 | 857 | 906 |
| 29.81 | 9,12-Octadecadienoic acid (Z,Z)-, methyl ester | MAINLIB | 112-63-0 | C19H34O2 | 294 | 8.41 | 833 | 849 |
| 29.81 | Linoleic acid ethyl ester | MAINLIB | 544-35-4 | C20H36O2 | 308 | 5.77 | 822 | 861 |

| Compound Structure | Delta | Hit Spectrum |
| --- | --- | --- |
|  |  |  |
|  |  |  |
|  |  |  |

| RT | Name | Library | Cas # | Molecular Formula | MolecularWeight | Probability | SI | RSI |
| --- | --- | --- | --- | --- | --- | --- | --- | --- |
| 29.90 | 9,12,15-Octadecatrienoic acid, 2,3-dihydroxypropyl ester, (Z,Z,Z)- | MAINLIB | 18465-99-1 | C21H36O4 | 352 | 36.12 | 828 | 839 |
| 29.90 | 8,11,14-Eicosatrienoic acid, (Z,Z,Z)- | MAINLIB | 1783-84-2 | C20H34O2 | 306 | 8.51 | 789 | 835 |
| 29.90 | 1-Heptatriacotanol | MAINLIB | 105794-58-9 | C37H76O | 536 | 7.52 | 786 | 795 |

| Compound Structure | Delta | Hit Spectrum |
| --- | --- | --- |
|  |  |  |
|  |  |  |
|  |  |  |

| RT | Name | Library | Cas # | Molecular Formula | MolecularWeight | Probability | SI | RSI |
| --- | --- | --- | --- | --- | --- | --- | --- | --- |
| 30.23 | 9,12-Octadecadienoic acid (Z,Z)-, trimethylsilyl ester | MAINLIB | 56259-07-5 | C21H40O2Si | 352 | 43.66 | 780 | 847 |
| 30.23 | 9-Octadecenoic acid, 2-[(trimethylsilyl)oxy]-1-[[(trimethylsilyl)oxy]methyl]ethyl ester | MAINLIB | 56554-42-8 | C27H56O4Si2 | 500 | 6.48 | 711 | 730 |
| 30.23 | 11,14-Eicosadienoic acid, trimethylsilyl ester | MAINLIB | NA | C23H44O2Si | 380 | 6.23 | 710 | 776 |

| Compound Structure | Delta | Hit Spectrum |
| --- | --- | --- |
|  |  |  |
|  |  |  |
|  |  |  |

| RT | Name | Library | Cas # | Molecular Formula | MolecularWeight | Probability | SI | RSI |
| --- | --- | --- | --- | --- | --- | --- | --- | --- |
| 30.35 | 9,12-Octadecadienoic acid (Z,Z)-, trimethylsilyl ester | MAINLIB | 56259-07-5 | C21H40O2Si | 352 | 57.49 | 784 | 839 |
| 30.35 | 11,14-Eicosadienoic acid, trimethylsilyl ester | MAINLIB | NA | C23H44O2Si | 380 | 6.55 | 705 | 760 |
| 30.35 | 9,12-Octadecadienal, dimethyl acetal | MAINLIB | 1599-51-5 | C20H38O2 | 310 | 5.53 | 701 | 738 |

| Compound Structure | Delta | Hit Spectrum |
| --- | --- | --- |
|  |  |  |
|  |  |  |
|  |  |  |

| RT | Name | Library | Cas # | Molecular Formula | MolecularWeight | Probability | SI | RSI |
| --- | --- | --- | --- | --- | --- | --- | --- | --- |
| 30.65 | 9,12-Octadecadienoic acid (Z,Z)-, trimethylsilyl ester | MAINLIB | 56259-07-5 | C21H40O2Si | 352 | 94.90 | 917 | 948 |
| 30.65 | 11,14-Eicosadienoic acid, trimethylsilyl ester | MAINLIB | NA | C23H44O2Si | 380 | 1.44 | 728 | 804 |
| 30.65 | 15-Isopropenyl-3-(trimethylsilyl)oxacyclopentadecan-2-one | MAINLIB | NA | C20H38O2Si | 338 | 0.65 | 708 | 714 |

| Compound Structure | Delta | Hit Spectrum |
| --- | --- | --- |
|  |  |  |
|  |  |  |
|  |  |  |

| RT | Name | Library | Cas # | Molecular Formula | MolecularWeight | Probability | SI | RSI |
| --- | --- | --- | --- | --- | --- | --- | --- | --- |
| 30.74 | à-Linolenic acid, trimethylsilyl ester | MAINLIB | 97844-13-8 | C21H38O2Si | 350 | 44.78 | 861 | 922 |
| 30.74 | Linolenic acid, trimethylsilyl ester | MAINLIB | NA | C21H38O2Si | 350 | 39.57 | 858 | 914 |
| 30.74 | trans-9-Octadecenoic acid, trimethylsilyl ester | MAINLIB | 96851-47-7 | C21H42O2Si | 354 | 2.50 | 747 | 799 |

| Compound Structure | Delta | Hit Spectrum |
| --- | --- | --- |
|  |  |  |
|  |  |  |
|  |  |  |

| RT | Name | Library | Cas # | Molecular Formula | MolecularWeight | Probability | SI | RSI |
| --- | --- | --- | --- | --- | --- | --- | --- | --- |
| 31.02 | Octadecanoic acid, trimethylsilyl ester | MAINLIB | 18748-91-9 | C21H44O2Si | 356 | 89.90 | 900 | 936 |
| 31.02 | Nonadecanoic acid, trimethylsilyl ester | MAINLIB | 74367-35-4 | C22H46O2Si | 370 | 1.83 | 726 | 763 |
| 31.02 | Tetradecanoic acid, trimethylsilyl ester | MAINLIB | 18603-17-3 | C17H36O2Si | 300 | 1.29 | 716 | 790 |

| Compound Structure | Delta | Hit Spectrum |
| --- | --- | --- |
|  |  |  |
|  |  |  |
|  |  |  |

| RT | Name | Library | Cas # | Molecular Formula | MolecularWeight | Probability | SI | RSI |
| --- | --- | --- | --- | --- | --- | --- | --- | --- |
| 31.75 | Tetradecane, 2,6,10-trimethyl- | MAINLIB | 14905-56-7 | C17H36 | 240 | 7.51 | 759 | 830 |
| 31.75 | Tetratetracontane | MAINLIB | 7098-22-8 | C44H90 | 618 | 6.05 | 754 | 810 |
| 31.75 | Heptacosane, 1-chloro- | MAINLIB | 62016-79-9 | C27H55Cl | 414 | 3.66 | 739 | 760 |

| Compound Structure | Delta | Hit Spectrum |
| --- | --- | --- |
|  |  |  |
|  |  |  |
|  |  |  |

| RT | Name | Library | Cas # | Molecular Formula | MolecularWeight | Probability | SI | RSI |
| --- | --- | --- | --- | --- | --- | --- | --- | --- |
| 33.12 | Heptacosane, 1-chloro- | MAINLIB | 62016-79-9 | C27H55Cl | 414 | 7.02 | 705 | 732 |
| 33.12 | Tetradecane, 2,6,10-trimethyl- | MAINLIB | 14905-56-7 | C17H36 | 240 | 5.93 | 701 | 806 |
| 33.12 | Octadecane, 3-ethyl-5-(2-ethylbutyl)- | MAINLIB | 55282-12-7 | C26H54 | 366 | 4.78 | 696 | 720 |

| Compound Structure | Delta | Hit Spectrum |
| --- | --- | --- |
|  |  |  |
|  |  |  |
|  |  |  |

| RT | Name | Library | Cas # | Molecular Formula | MolecularWeight | Probability | SI | RSI |
| --- | --- | --- | --- | --- | --- | --- | --- | --- |
| 33.38 | 11-Eicosenoic acid, trimethylsilyl ester | MAINLIB | NA | C23H46O2Si | 382 | 70.30 | 815 | 894 |
| 33.38 | trans-9-Octadecenoic acid, trimethylsilyl ester | MAINLIB | 96851-47-7 | C21H42O2Si | 354 | 4.66 | 708 | 815 |
| 33.38 | á-D-Galactopyranoside, methyl 2,3-bis-O-(trimethylsilyl)-, cyclic methylboronate | MAINLIB | 56211-08-6 | C14H31BO6Si2 | 362 | 3.94 | 704 | 733 |

| Compound Structure | Delta | Hit Spectrum |
| --- | --- | --- |
|  |  |  |
|  |  |  |
|  |  |  |

| RT | Name | Library | Cas # | Molecular Formula | MolecularWeight | Probability | SI | RSI |
| --- | --- | --- | --- | --- | --- | --- | --- | --- |
| 33.72 | Eicosanoic acid, trimethylsilyl ester | MAINLIB | 55530-70-6 | C23H48O2Si | 384 | 17.80 | 704 | 745 |
| 33.72 | Octadecanoic acid, trimethylsilyl ester | MAINLIB | 18748-91-9 | C21H44O2Si | 356 | 11.14 | 690 | 840 |
| 33.72 | Nonadecanoic acid, trimethylsilyl ester | MAINLIB | 74367-35-4 | C22H46O2Si | 370 | 7.87 | 680 | 800 |

| Compound Structure | Delta | Hit Spectrum |
| --- | --- | --- |
|  |  |  |
|  |  |  |
|  |  |  |

| RT | Name | Library | Cas # | Molecular Formula | MolecularWeight | Probability | SI | RSI |
| --- | --- | --- | --- | --- | --- | --- | --- | --- |
| 35.11 | 1,2-Benzenedicarboxylic acid, mono(2-ethylhexyl) ester | MAINLIB | 4376-20-9 | C16H22O4 | 278 | 17.42 | 715 | 784 |
| 35.11 | 9-(2',2'-Dimethylpropanoilhydrazono)-3,6-dichloro-2,7-bis-[2-(diethylamino)-ethoxy]fluorene | MAINLIB | NA | C30H42Cl2N4O3 | 576 | 11.95 | 704 | 785 |
| 35.11 | 1,2-Benzenedicarboxylic acid, diisooctyl ester | MAINLIB | 27554-26-3 | C24H38O4 | 390 | 9.63 | 699 | 807 |

| Compound Structure | Delta | Hit Spectrum |
| --- | --- | --- |
|  |  |  |
|  |  |  |
|  |  |  |

| RT | Name | Library | Cas # | Molecular Formula | MolecularWeight | Probability | SI | RSI |
| --- | --- | --- | --- | --- | --- | --- | --- | --- |
| 35.75 | Hexadecanoic acid, 2,3-bis[(trimethylsilyl)oxy]propyl ester | MAINLIB | 1188-74-5 | C25H54O4Si2 | 474 | 93.13 | 865 | 888 |
| 35.75 | 2-Monopalmitin trimethylsilyl ether | MAINLIB | 53212-97-8 | C25H54O4Si2 | 474 | 2.23 | 700 | 723 |
| 35.75 | Hexadecanoic acid, 3-[(trimethylsilyl)oxy]propyl ester | MAINLIB | 56630-48-9 | C22H46O3Si | 386 | 1.48 | 688 | 727 |

| Compound Structure | Delta | Hit Spectrum |
| --- | --- | --- |
|  |  |  |
|  |  |  |
|  |  |  |

| RT | Name | Library | Cas # | Molecular Formula | MolecularWeight | Probability | SI | RSI |
| --- | --- | --- | --- | --- | --- | --- | --- | --- |
| 36.23 | Docosanoic acid, trimethylsilyl ester | MAINLIB | 74367-36-5 | C25H52O2Si | 412 | 76.38 | 822 | 878 |
| 36.23 | Octadecanoic acid, trimethylsilyl ester | MAINLIB | 18748-91-9 | C21H44O2Si | 356 | 3.11 | 691 | 834 |
| 36.23 | Tetradecanoic acid, trimethylsilyl ester | MAINLIB | 18603-17-3 | C17H36O2Si | 300 | 2.51 | 686 | 777 |

| Compound Structure | Delta | Hit Spectrum |
| --- | --- | --- |
|  |  |  |
|  |  |  |
|  |  |  |

| RT | Name | Library | Cas # | Molecular Formula | MolecularWeight | Probability | SI | RSI |
| --- | --- | --- | --- | --- | --- | --- | --- | --- |
| 37.43 | à-D-Glucopyranoside, methyl 2-(acetylamino)-2-deoxy-3-O-(trimethylsilyl)-, cyclic methylboronate | MAINLIB | 54477-01-9 | C13H26BNO6Si | 331 | 23.90 | 706 | 747 |
| 37.43 | á-D-Galactopyranoside, methyl 2,3-bis-O-(trimethylsilyl)-, cyclic methylboronate | MAINLIB | 56211-08-6 | C14H31BO6Si2 | 362 | 19.26 | 701 | 734 |
| 37.43 | á-D-Galactopyranoside, methyl 2,6-bis-O-(trimethylsilyl)-, cyclic methylboronate | MAINLIB | 56211-06-4 | C14H31BO6Si2 | 362 | 14.75 | 694 | 731 |

| Compound Structure | Delta | Hit Spectrum |
| --- | --- | --- |
|  |  |  |
|  |  |  |
|  |  |  |

| RT | Name | Library | Cas # | Molecular Formula | MolecularWeight | Probability | SI | RSI |
| --- | --- | --- | --- | --- | --- | --- | --- | --- |
| 37.78 | 9-Octadecenoic acid, 2-[(trimethylsilyl)oxy]-1-[[(trimethylsilyl)oxy]methyl]ethyl ester | MAINLIB | 56554-42-8 | C27H56O4Si2 | 500 | 78.15 | 815 | 837 |
| 37.78 | 1-Monolinoleoylglycerol trimethylsilyl ether | MAINLIB | 54284-45-6 | C27H54O4Si2 | 498 | 6.82 | 727 | 733 |
| 37.78 | 1-Monooleoylglycerol trimethylsilyl ether | MAINLIB | 54284-47-8 | C27H56O4Si2 | 500 | 5.36 | 721 | 765 |

| Compound Structure | Delta | Hit Spectrum |
| --- | --- | --- |
|  |  |  |
|  |  |  |
|  |  |  |

| RT | Name | Library | Cas # | Molecular Formula | MolecularWeight | Probability | SI | RSI |
| --- | --- | --- | --- | --- | --- | --- | --- | --- |
| 37.88 | 9-Octadecenoic acid, 2-[(trimethylsilyl)oxy]-1-[[(trimethylsilyl)oxy]methyl]ethyl ester | MAINLIB | 56554-42-8 | C27H56O4Si2 | 500 | 55.98 | 768 | 802 |
| 37.88 | 1-Monolinoleoylglycerol trimethylsilyl ether | MAINLIB | 54284-45-6 | C27H54O4Si2 | 498 | 8.66 | 701 | 725 |
| 37.88 | à-D-Glucopyranoside, methyl 2-(acetylamino)-2-deoxy-3-O-(trimethylsilyl)-, cyclic methylboronate | MAINLIB | 54477-01-9 | C13H26BNO6Si | 331 | 5.94 | 690 | 714 |

| Compound Structure | Delta | Hit Spectrum |
| --- | --- | --- |
|  |  |  |
|  |  |  |
|  |  |  |

| RT | Name | Library | Cas # | Molecular Formula | MolecularWeight | Probability | SI | RSI |
| --- | --- | --- | --- | --- | --- | --- | --- | --- |
| 38.58 | á-D-Galactopyranoside, methyl 2,3-bis-O-(trimethylsilyl)-, cyclic methylboronate | MAINLIB | 56211-08-6 | C14H31BO6Si2 | 362 | 14.25 | 688 | 723 |
| 38.58 | à-D-Glucopyranoside, methyl 2-(acetylamino)-2-deoxy-3-O-(trimethylsilyl)-, cyclic methylboronate | MAINLIB | 54477-01-9 | C13H26BNO6Si | 331 | 13.70 | 687 | 727 |
| 38.58 | á-D-Galactopyranoside, methyl 2,6-bis-O-(trimethylsilyl)-, cyclic methylboronate | MAINLIB | 56211-06-4 | C14H31BO6Si2 | 362 | 9.12 | 675 | 713 |

| Compound Structure | Delta | Hit Spectrum |
| --- | --- | --- |
|  |  |  |
|  |  |  |
|  |  |  |

| RT | Name | Library | Cas # | Molecular Formula | MolecularWeight | Probability | SI | RSI |
| --- | --- | --- | --- | --- | --- | --- | --- | --- |
| 42.08 | 4-Azido-2-nitrobutyric acid, 2,6-di-t-butyl-4-methoxyphenyl ester | MAINLIB | 113719-06-5 | C19H28N4O5 | 392 | 12.51 | 588 | 645 |
| 42.08 | Androstane, 17-(2(5H)-oxofuran-4-yl)-3-(t-butyldimethylsilyloxy)-14-(trimethylsilyloxy)- | MAINLIB | NA | C32H56O4Si2 | 560 | 12.03 | 587 | 589 |
| 42.08 | à-Tocopherol (vitamin E), trimethysilyl derivative | MAINLIB | 2733-26-8 | C32H58O2Si | 502 | 11.09 | 585 | 645 |

| Compound Structure | Delta | Hit Spectrum |
| --- | --- | --- |
|  |  |  |
|  |  |  |
|  |  |  |

| RT | Name | Library | Cas # | Molecular Formula | MolecularWeight | Probability | SI | RSI |
| --- | --- | --- | --- | --- | --- | --- | --- | --- |
| 43.34 | Campesterol | MAINLIB | 474-62-4 | C28H48O | 400 | 18.72 | 714 | 726 |
| 43.34 | 3à-(Trimethylsiloxy)cholest-5-ene | MAINLIB | 16134-40-0 | C30H54OSi | 458 | 9.65 | 696 | 834 |
| 43.34 | Ergost-5-en-3-ol, acetate, (3á,24R)- | MAINLIB | NA | C30H50O2 | 442 | 9.65 | 696 | 799 |

| Compound Structure | Delta | Hit Spectrum |
| --- | --- | --- |
|  |  |  |
|  |  |  |
|  |  |  |

| RT | Name | Library | Cas # | Molecular Formula | MolecularWeight | Probability | SI | RSI |
| --- | --- | --- | --- | --- | --- | --- | --- | --- |
| 43.65 | Stigmasterol trimethylsilyl ether | MAINLIB | 14030-29-6 | C32H56OSi | 484 | 56.58 | 783 | 795 |
| 43.65 | Stigmastan-6,22-dien, 3,5-dedihydro- | MAINLIB | 107304-12-1 | C29H46 | 394 | 7.88 | 711 | 811 |
| 43.65 | Ergosta-5,22-dien-3-ol, acetate, (3á,22E)- | MAINLIB | 2458-53-9 | C30H48O2 | 440 | 6.35 | 706 | 753 |

| Compound Structure | Delta | Hit Spectrum |
| --- | --- | --- |
|  |  |  |
|  |  |  |
|  |  |  |

| RT | Name | Library | Cas # | Molecular Formula | MolecularWeight | Probability | SI | RSI |
| --- | --- | --- | --- | --- | --- | --- | --- | --- |
| 44.27 | á-Sitosterol trimethylsilyl ether | MAINLIB | 2625-46-9 | C32H58OSi | 486 | 59.31 | 797 | 799 |
| 44.27 | 3à-(Trimethylsiloxy)cholest-5-ene | MAINLIB | 16134-40-0 | C30H54OSi | 458 | 7.89 | 723 | 821 |
| 44.27 | ç-Sitosterol | MAINLIB | 83-47-6 | C29H50O | 414 | 5.57 | 713 | 722 |

| Compound Structure | Delta | Hit Spectrum |
| --- | --- | --- |
|  |  |  |
|  |  |  |
|  |  |  |

| RT | Name | Library | Cas # | Molecular Formula | MolecularWeight | Probability | SI | RSI |
| --- | --- | --- | --- | --- | --- | --- | --- | --- |
| 44.84 | à-D-Glucopyranoside, methyl 2-(acetylamino)-2-deoxy-3-O-(trimethylsilyl)-, cyclic methylboronate | MAINLIB | 54477-01-9 | C13H26BNO6Si | 331 | 16.81 | 703 | 764 |
| 44.84 | 1-Monolinoleoylglycerol trimethylsilyl ether | MAINLIB | 54284-45-6 | C27H54O4Si2 | 498 | 16.81 | 703 | 765 |
| 44.84 | 9,12,15-Octadecatrienoic acid, 2-[(trimethylsilyl)oxy]-1-[[(trimethylsilyl)oxy]methyl]ethyl ester, (Z,Z,Z)- | MAINLIB | 55521-23-8 | C27H52O4Si2 | 496 | 12.87 | 696 | 803 |

| Compound Structure | Delta | Hit Spectrum |
| --- | --- | --- |
|  |  |  |
|  |  |  |
|  |  |  |

| RT | Name | Library | Cas # | Molecular Formula | MolecularWeight | Probability | SI | RSI |
| --- | --- | --- | --- | --- | --- | --- | --- | --- |
| 45.04 | 1-Monolinoleoylglycerol trimethylsilyl ether | MAINLIB | 54284-45-6 | C27H54O4Si2 | 498 | 26.57 | 719 | 767 |
| 45.04 | à-D-Glucopyranoside, methyl 2-(acetylamino)-2-deoxy-3-O-(trimethylsilyl)-, cyclic methylboronate | MAINLIB | 54477-01-9 | C13H26BNO6Si | 331 | 12.90 | 700 | 759 |
| 45.04 | 9,12,15-Octadecatrienoic acid, 2-[(trimethylsilyl)oxy]-1-[[(trimethylsilyl)oxy]methyl]ethyl ester, (Z,Z,Z)- | MAINLIB | 55521-23-8 | C27H52O4Si2 | 496 | 10.90 | 696 | 783 |

| Compound Structure | Delta | Hit Spectrum |
| --- | --- | --- |
|  |  |  |
|  |  |  |
|  |  |  |

| RT | Name | Library | Cas # | Molecular Formula | MolecularWeight | Probability | SI | RSI |
| --- | --- | --- | --- | --- | --- | --- | --- | --- |
| 45.62 | 1-Monolinoleoylglycerol trimethylsilyl ether | MAINLIB | 54284-45-6 | C27H54O4Si2 | 498 | 58.55 | 728 | 775 |
| 45.62 | 9,12,15-Octadecatrienoic acid, 2-[(trimethylsilyl)oxy]-1-[[(trimethylsilyl)oxy]methyl]ethyl ester, (Z,Z,Z)- | MAINLIB | 55521-23-8 | C27H52O4Si2 | 496 | 15.64 | 696 | 781 |
| 45.62 | à-D-Glucopyranoside, methyl 2-(acetylamino)-2-deoxy-3-O-(trimethylsilyl)-, cyclic methylboronate | MAINLIB | 54477-01-9 | C13H26BNO6Si | 331 | 11.67 | 688 | 748 |
