# Supplementary material for: Tetrastigma hemsleyanum (Sanyeqing) root extracts evoke S phase arrest while inhibiting the migration and invasion of human pancreatic cancer PANC-1 cells
Source: BMC Complement Med Ther. 2024 Mar 27;24:133. doi: 10.1186/s12906-024-04425-1 (PMC10967071; doi:10.1186/s12906-024-04425-1)
Supplement: Supplementary file 2 — Supplementary Material 2 [file 12906_2024_4425_MOESM2_ESM.docx]

**Table S1** Distribution of cell cycle phases of PANC-1 cells (means ± SD, n=3)

**P* < 0.05

***P* < 0.01 vs. Control

| Treatment | | Cell-cycle distribution(%) | | |
| --- | --- | --- | --- | --- |
|  |  | G_0_/G_1_ | S | G_2_M |
| Control (0.1% DMSO) | | 35.1±3.84 | 39.9±3.79 | 26.7±2.64 |
| EFT concentration | 50 µg/mL | 33.1±3.50 | 41.5±4.83 | 25.1±0.78 |
|  | 100 µg/mL | 27.5±2.87 | 56.4±4.33** | 23.7±5.57 |
|  | 200 µg/mL | 22.6±1.57* | 75.8±3.57** | 9.2±6.48* |

**Table S2** EFT inhibits cell proliferation in PANC-1 cell

**P* < 0.05

***P* < 0.01 vs. Control

| Time | EFT (μg/mL) | | | | | |
| --- | --- | --- | --- | --- | --- | --- |
|  | 0 | 25 | 50 | 100 | 200 | 400 |
| 12 h | 100.00% | (93.01±5.90)% | (92.37±3.32)%* | (86.95±2.92)%* | (69.74±4.21)%** | (63.64±9.82)%** |
| 24 h | 100.00% | (82.44±11.07)% | (74.87±4.28)%** | (66.05±5.22)%** | (42.16±3.31)%** | (34.86±1.46)%** |
| 48 h | 100.00% | (80.62±9.35)%* | (60.18±7.28)%* | (49.14±4.06)%** | (31.71±3.96)%** | (23.37±4.65)%** |
| 72 h | 100.00% | (70.59±10.29)%* | (61.67±1.59)%** | (43.81±5.12)%** | (27.76±2.76)%** | (22.19±1.56)%** |

**Table S3** EFT inhibits cell proliferation in Bxpc-3 cell

**P* < 0.05

***P* < 0.01 vs. Control

| Time | EFT (μg/mL) | | | | | |
| --- | --- | --- | --- | --- | --- | --- |
|  | 0 | 25 | 50 | 100 | 200 | 400 |
| 12 h | 100.00% | (93.88±5.90)% | (88.11±3.32)%* | (80.30±2.92)%** | (73.48±4.21)%** | (74.78±9.81)%** |
| 24 h | 100.00% | (87.37±11.07)% | (76.18±4.28)%* | (69.51±5.21)%* | (65.31±3.31)%** | (58.83±1.46)%** |
| 48 h | 100.00% | (88.08±9.35)% | (74.88±7.28)%* | (65.88±4.06)%* | (62.88±3.95)%** | (52.57±4.65)%** |
| 72 h | 100.00% | (82.44±10.29)% | (74.87±1.59)%** | (66.05±5.11)%** | (42.16±2.76)%** | (34.86±1.55)%** |

**
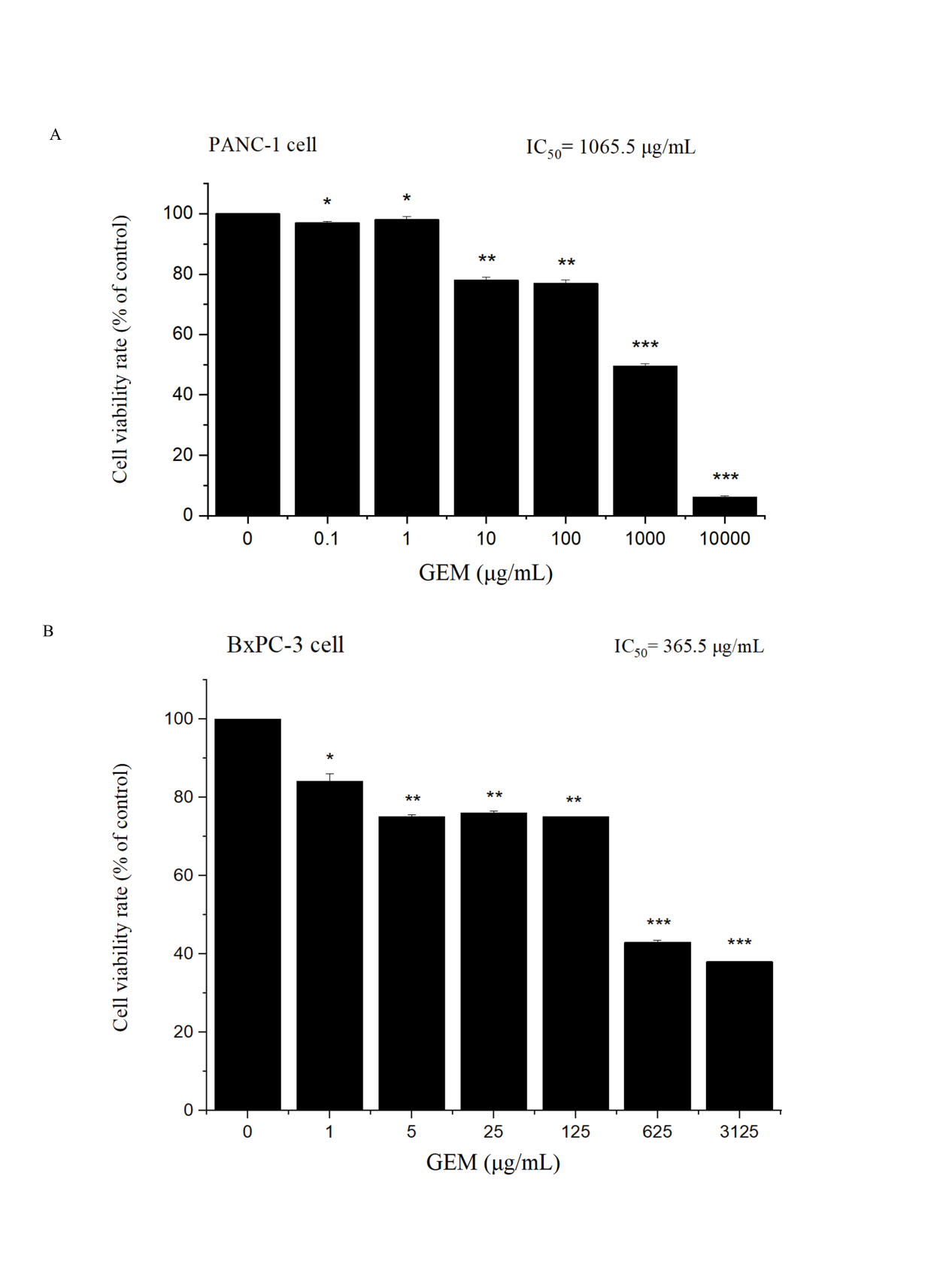
 Figure S1** Gemcitabine inhibits cell proliferation in BxPC-3 and PANC-1 cells. PANC-1 cell (A) and BxPC-3 cell (B) viability inhibitory rate determined by MTT after incubation for 24h. **P* < 0.05, ***P* < 0.01, ****P* < 0.001.


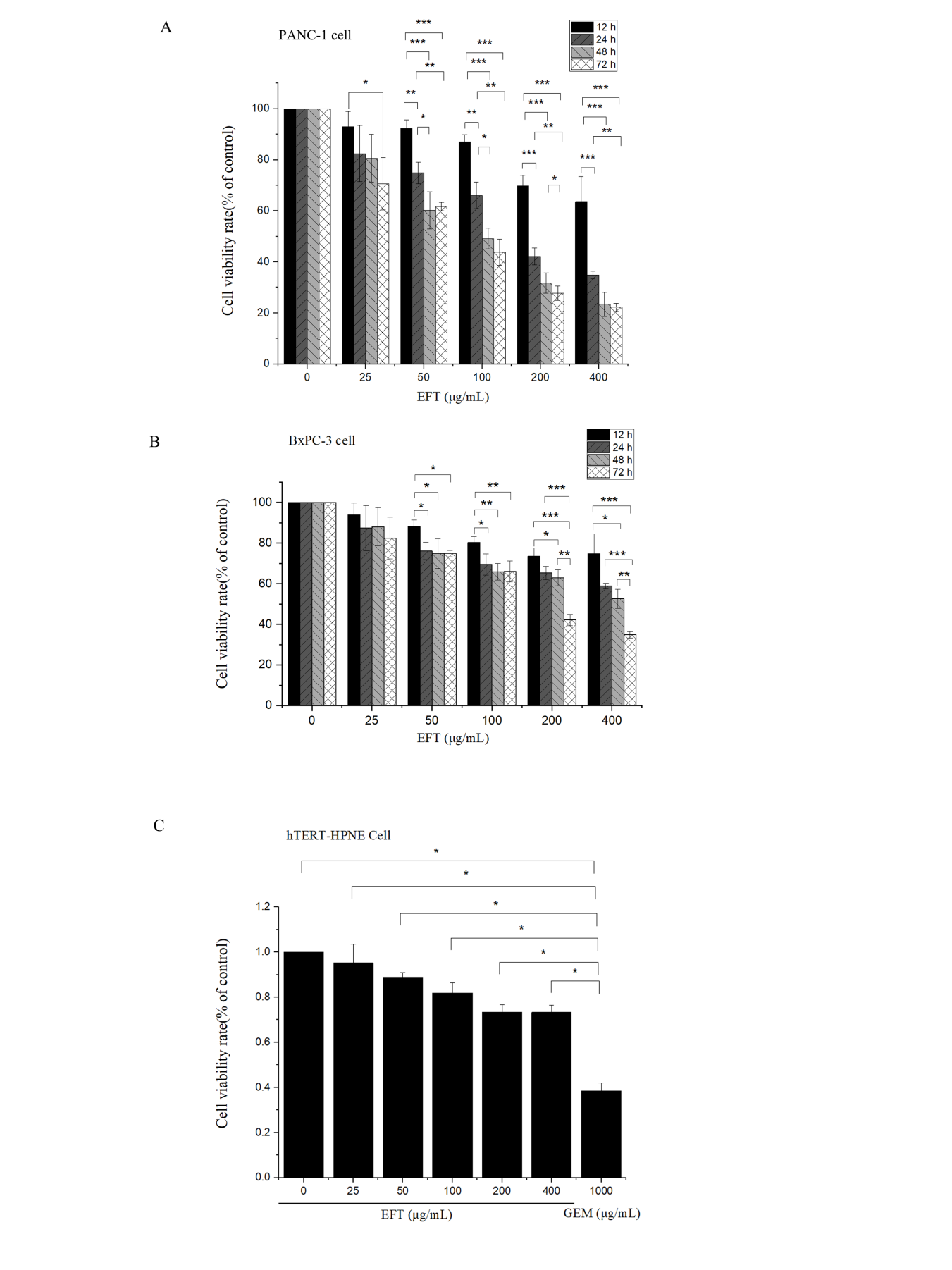


**Figure S2** EFT inhibits cell proliferation in BxPC-3 and PANC-1 cells. PANC-1 cell (A) and BxPC-3 cell (B) viability inhibitory rate determined by MTT after incubation for 12 h, 24 h, 48 h, and 72 h. (C) hTERT-HPNE cells were used as normal control. Data represent the mean ± SD of three replicates and three independent experiments. **P* < 0.05, ***P* < 0.01, ****P* < 0.001, compared with control (0.1% DMSO only). Gemcitabine (1000 μg/mL) were used as the positive control.
