# Supplementary material for: Tetrastigma hemsleyanum (Sanyeqing) root extracts evoke S phase arrest while inhibiting the migration and invasion of human pancreatic cancer PANC-1 cells
Source: BMC Complement Med Ther. 2024 Mar 27;24:133. doi: 10.1186/s12906-024-04425-1 (PMC10967071; doi:10.1186/s12906-024-04425-1)
Supplement: Supplementary file 3 — Supplementary Material 3 [file 12906_2024_4425_MOESM3_ESM.pdf]

Croppedblots in main paper

Croppedblots in supplementary information

Figure 3C

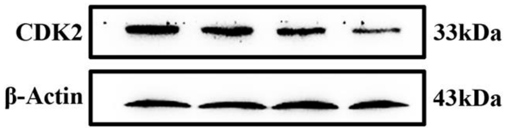

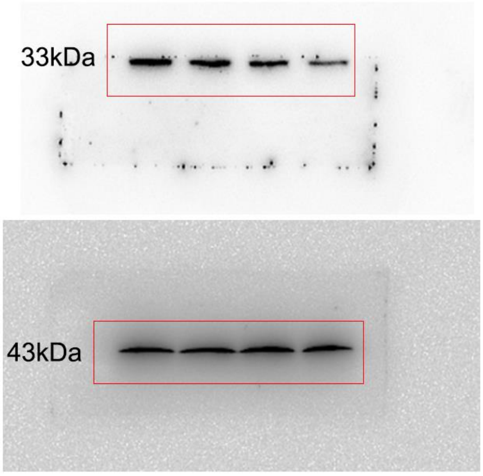

Figure 3E

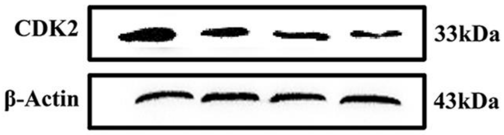

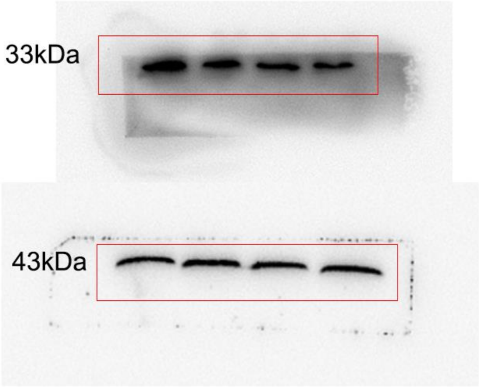

Croppedblots in supplementary information

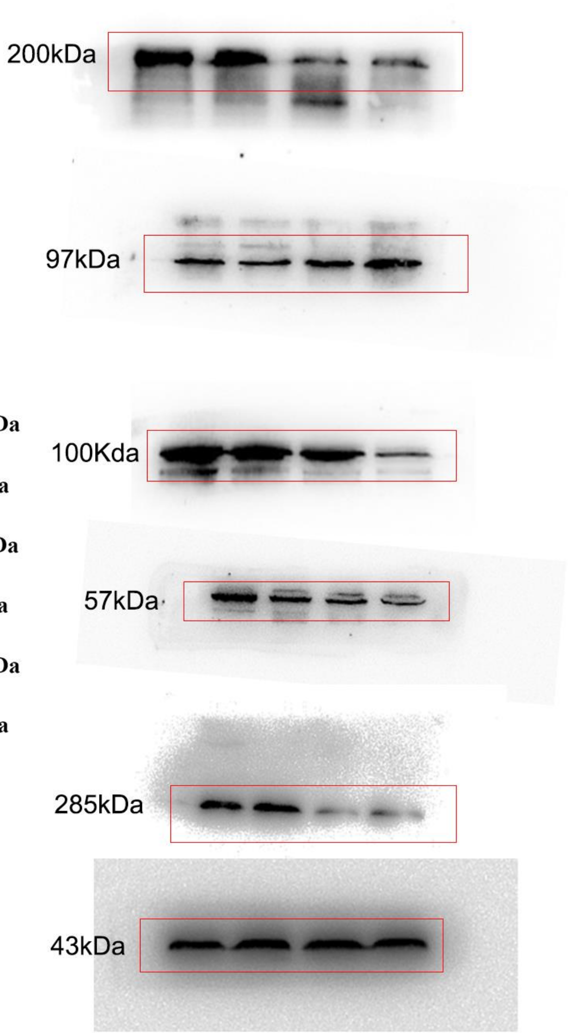

Croppedblots in main paper

Figure5A

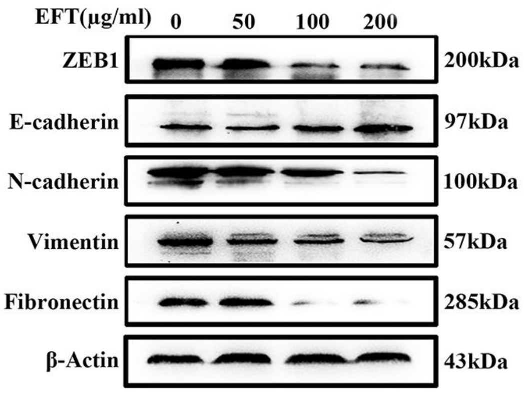

Croppedblots in main paper

Figure5C

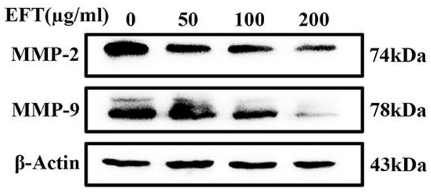

Croppedblots in supplementary information

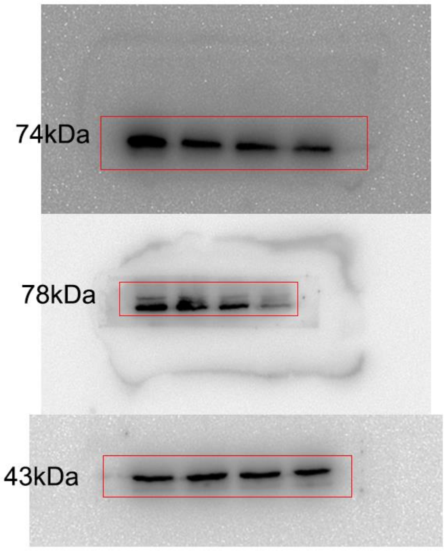

Figure 3C

CDK2

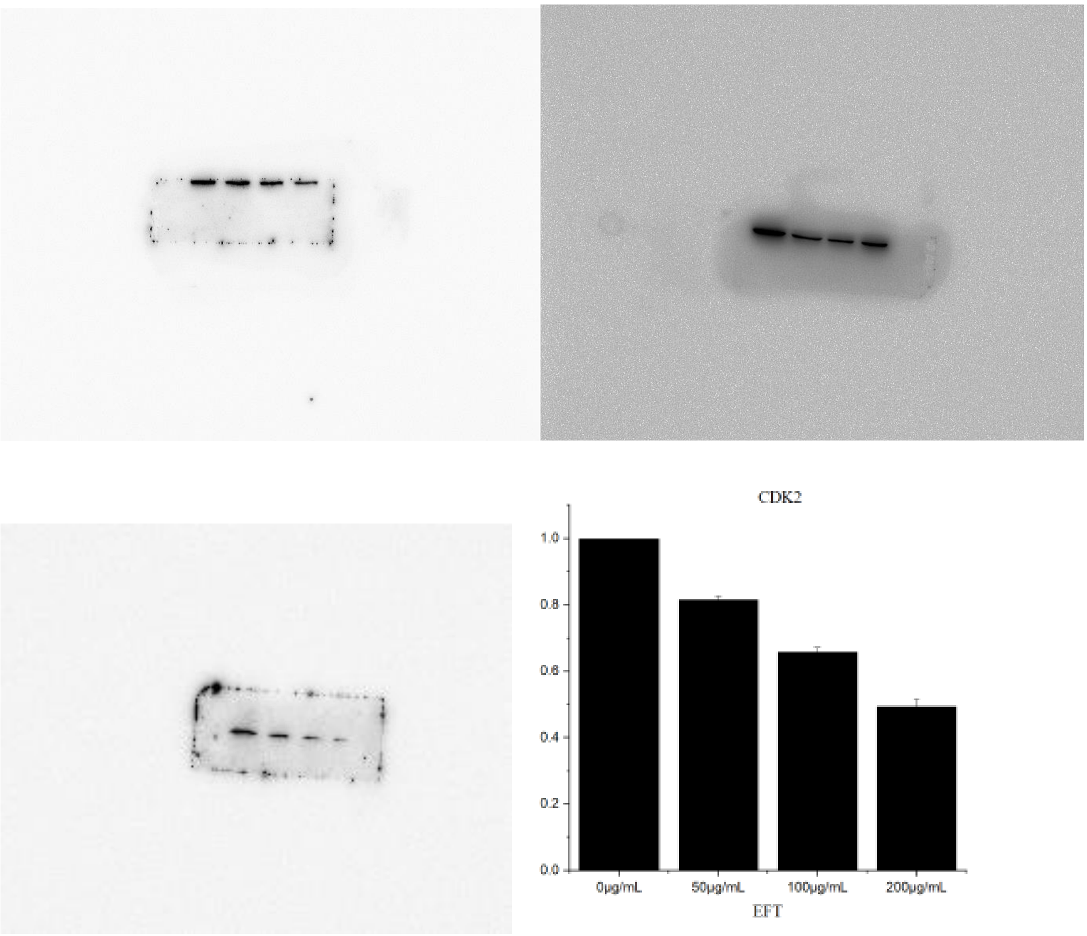

Figure 3C

β-actin

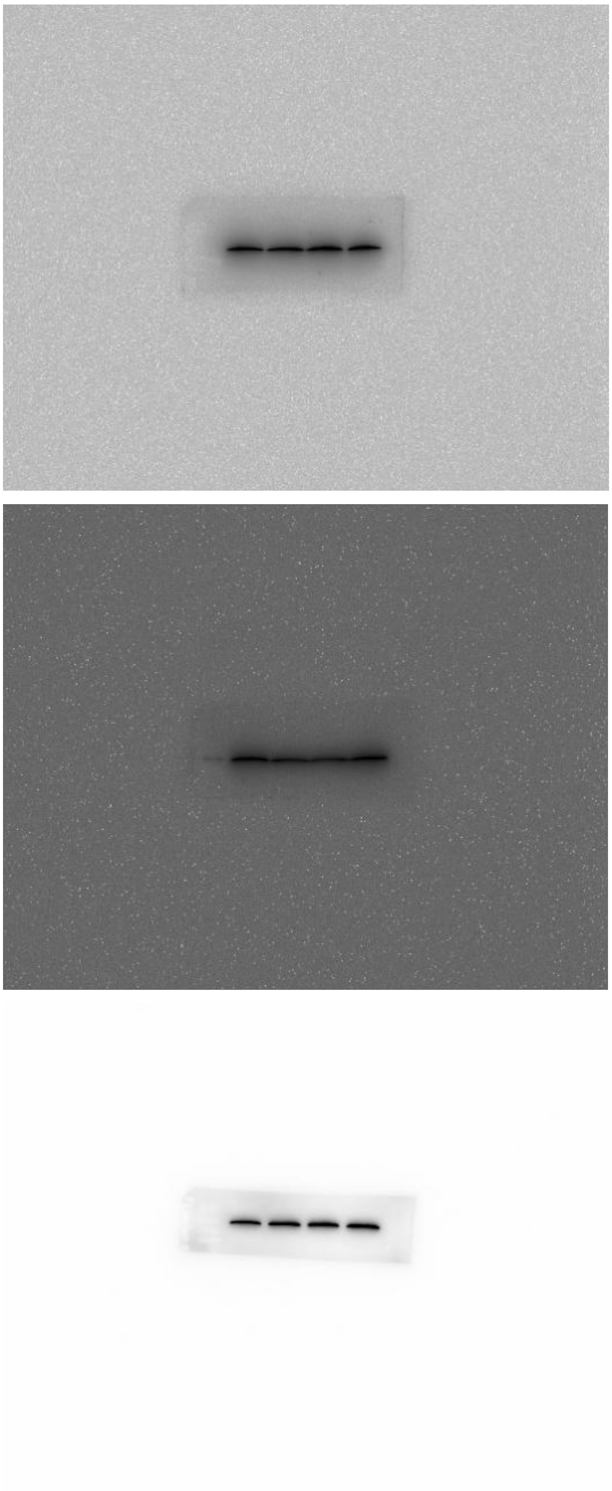

Figure 3E

CDK2

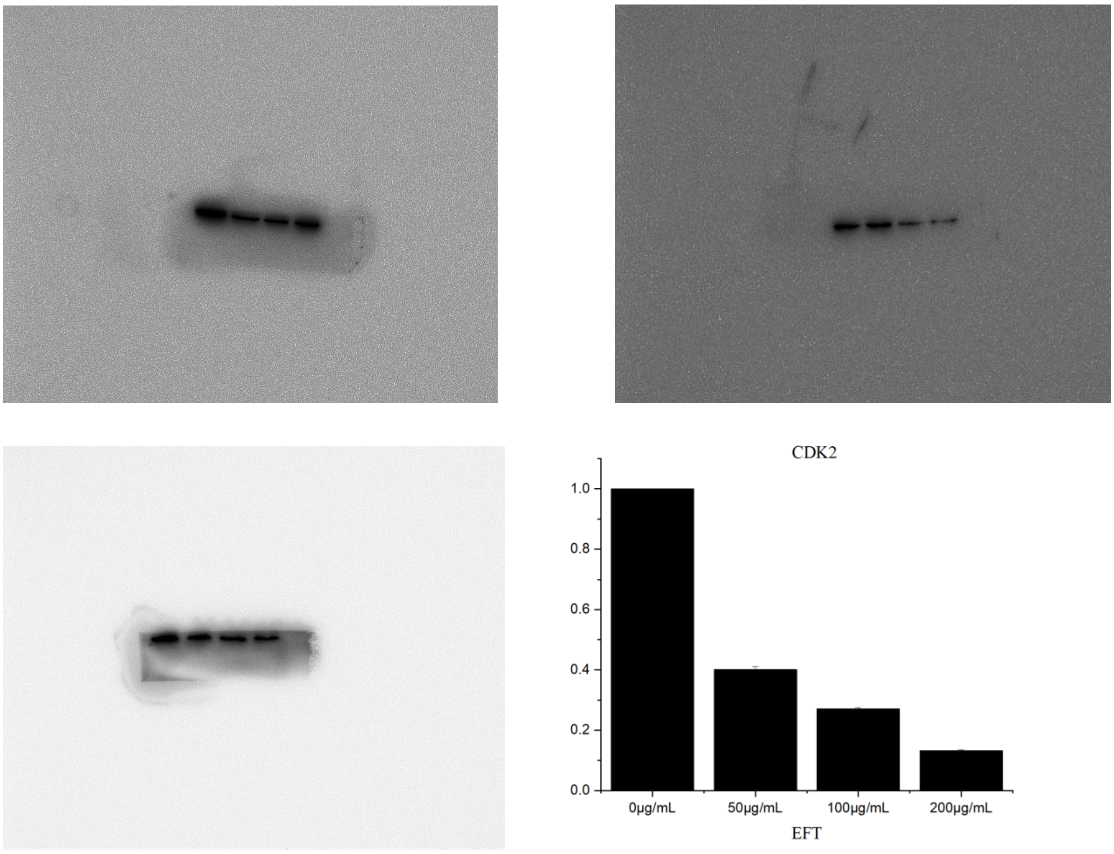

Figure 3E

β-actin

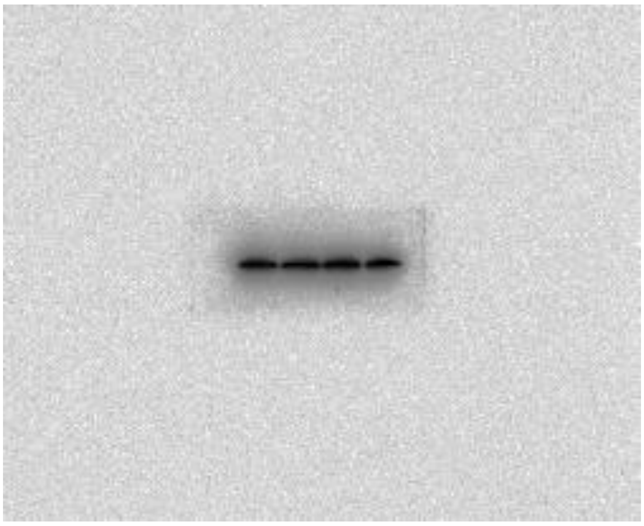

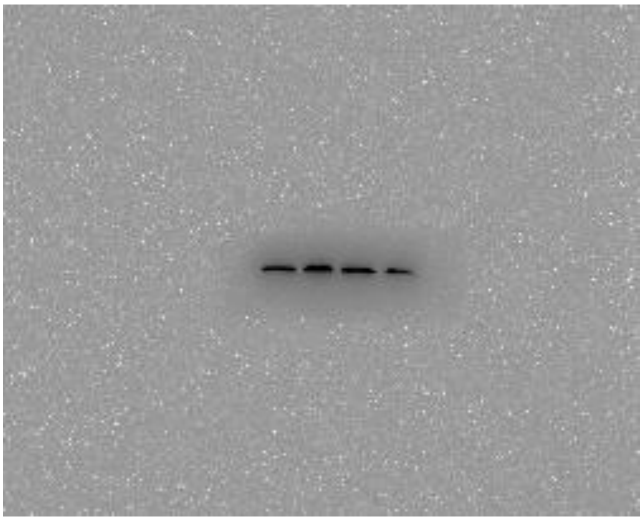

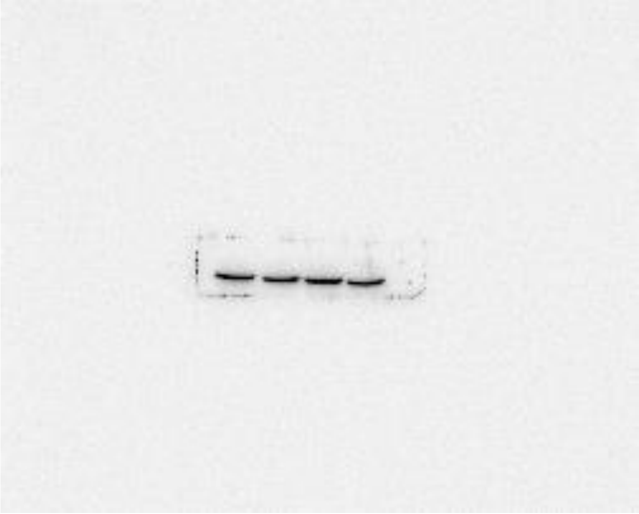

Figure 5A

E-cadherin

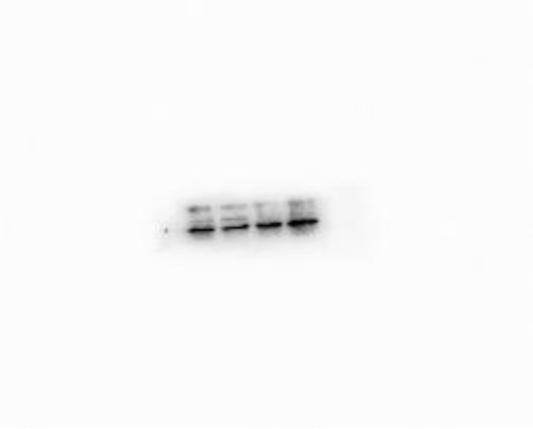

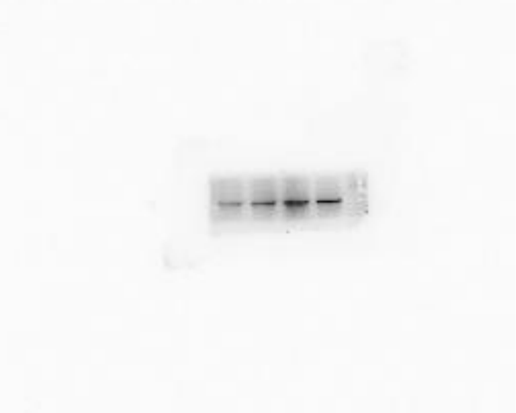

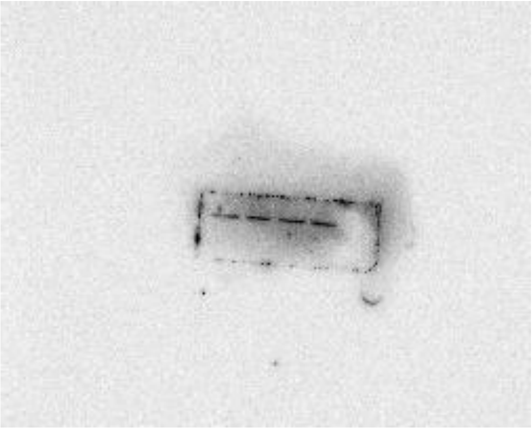

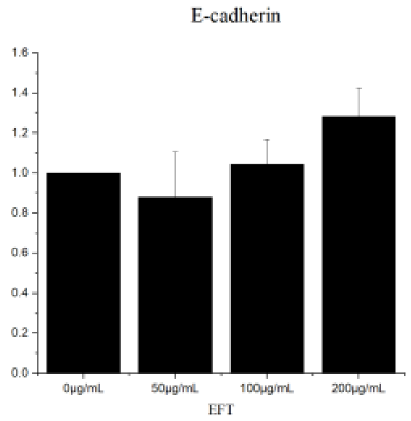

Figure 5A

Fibronectin

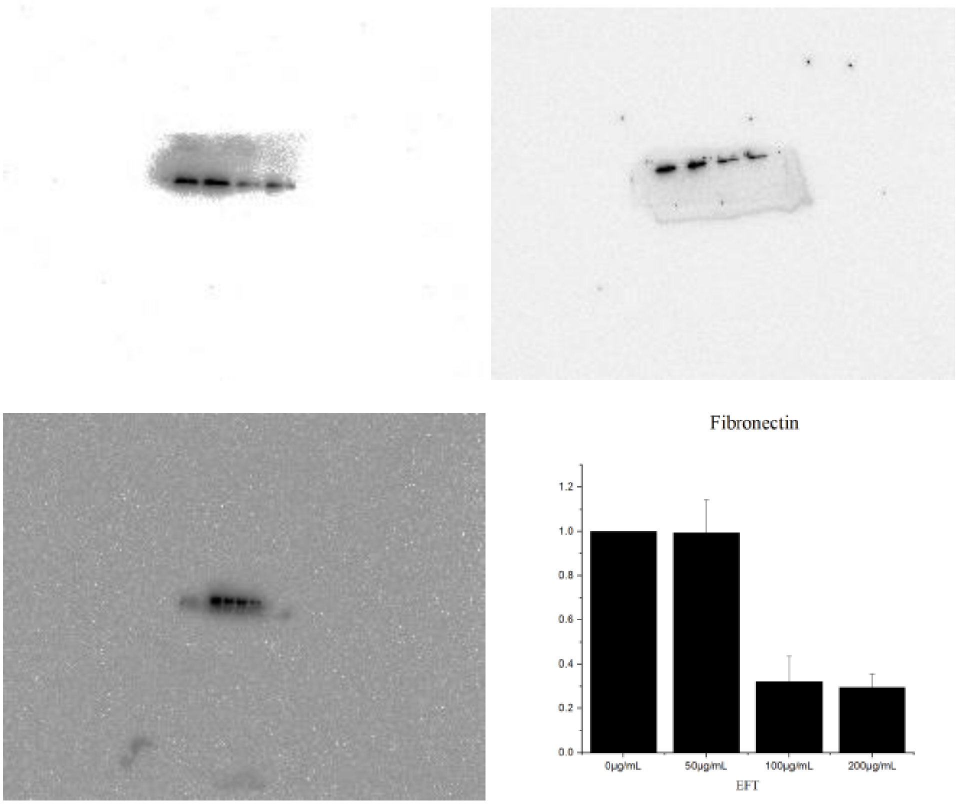

Figure 5A

N-cadherin

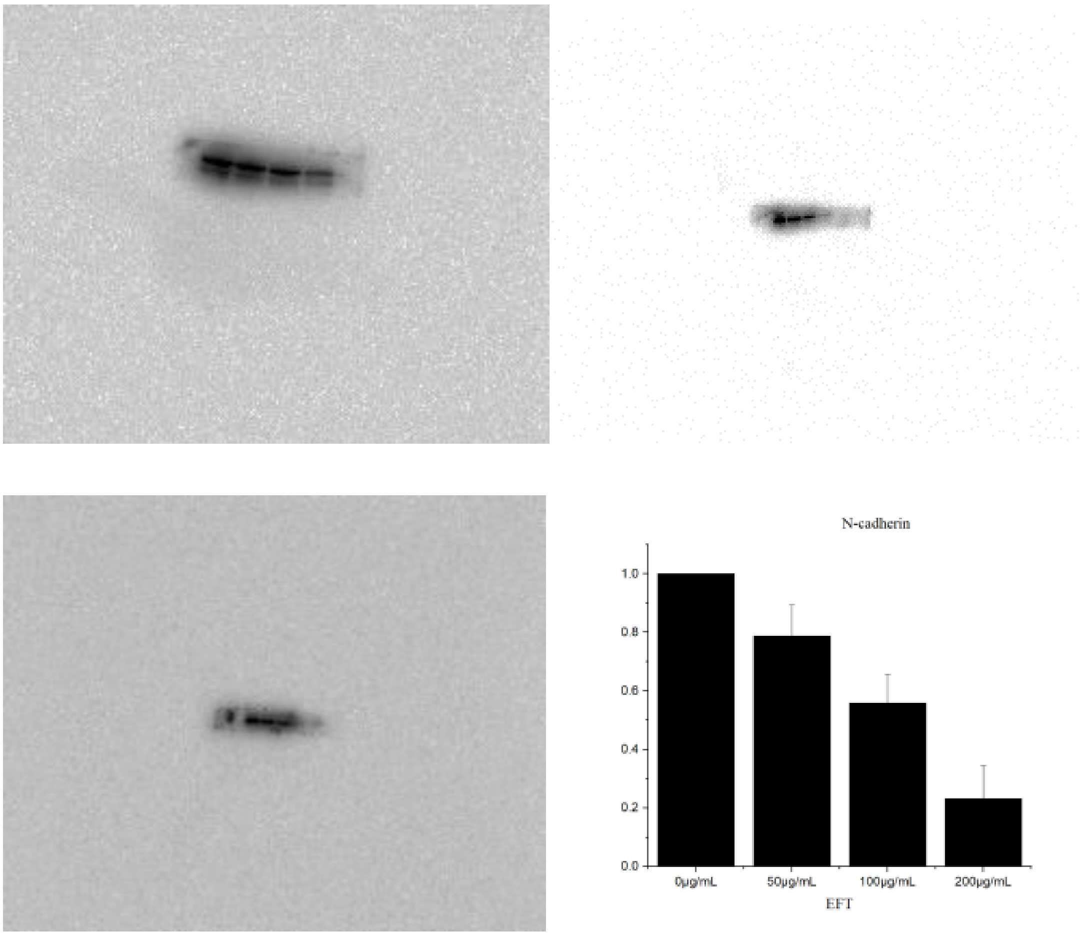

Figure 5A

Vimentin

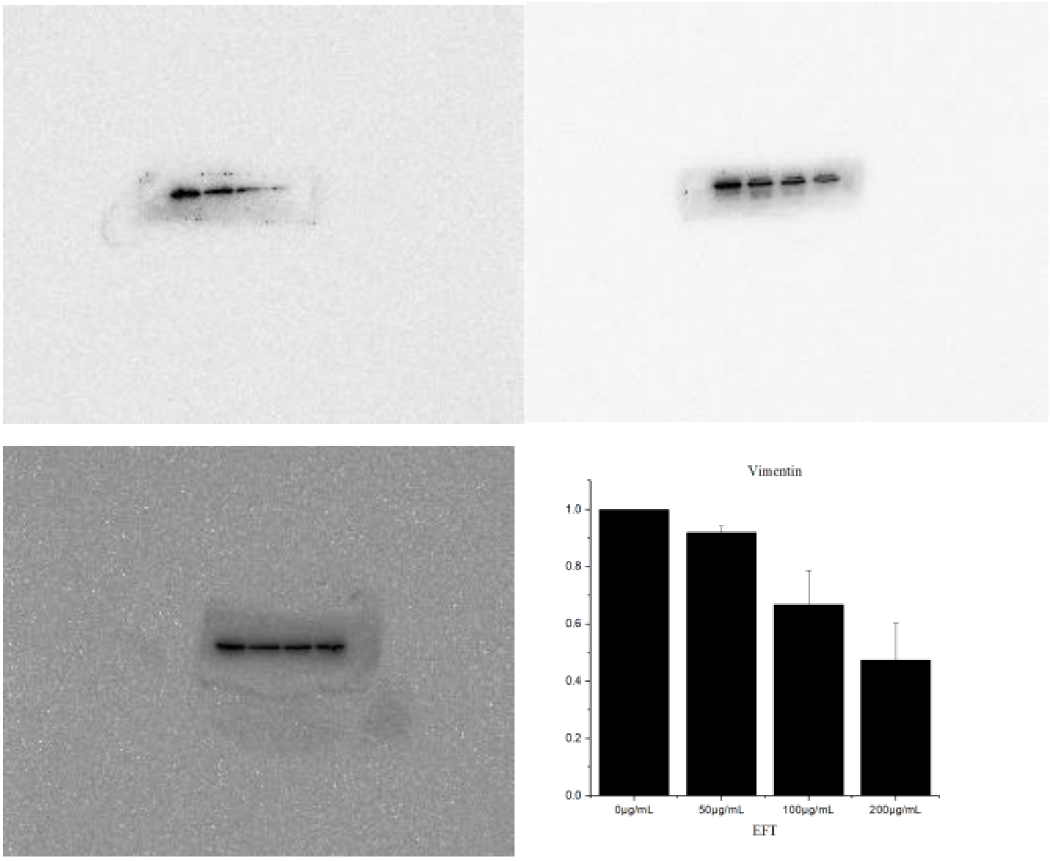

Figure 5A

ZEB1

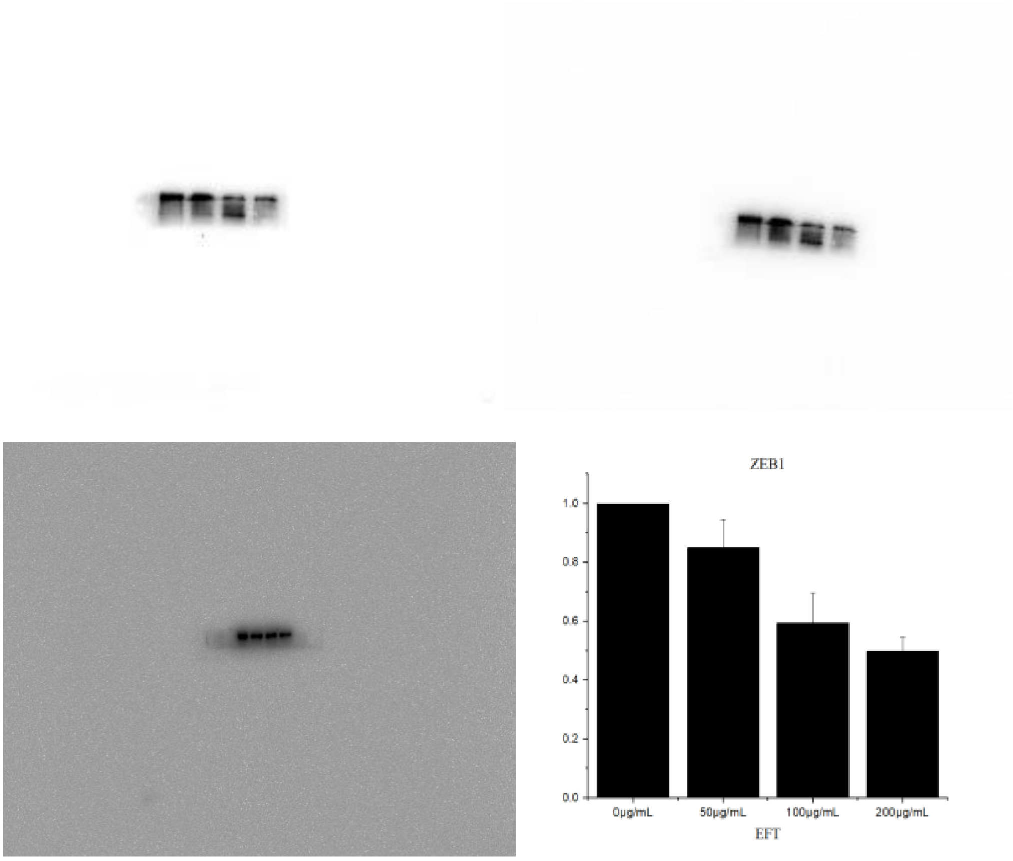

Figure 5A

β-actin

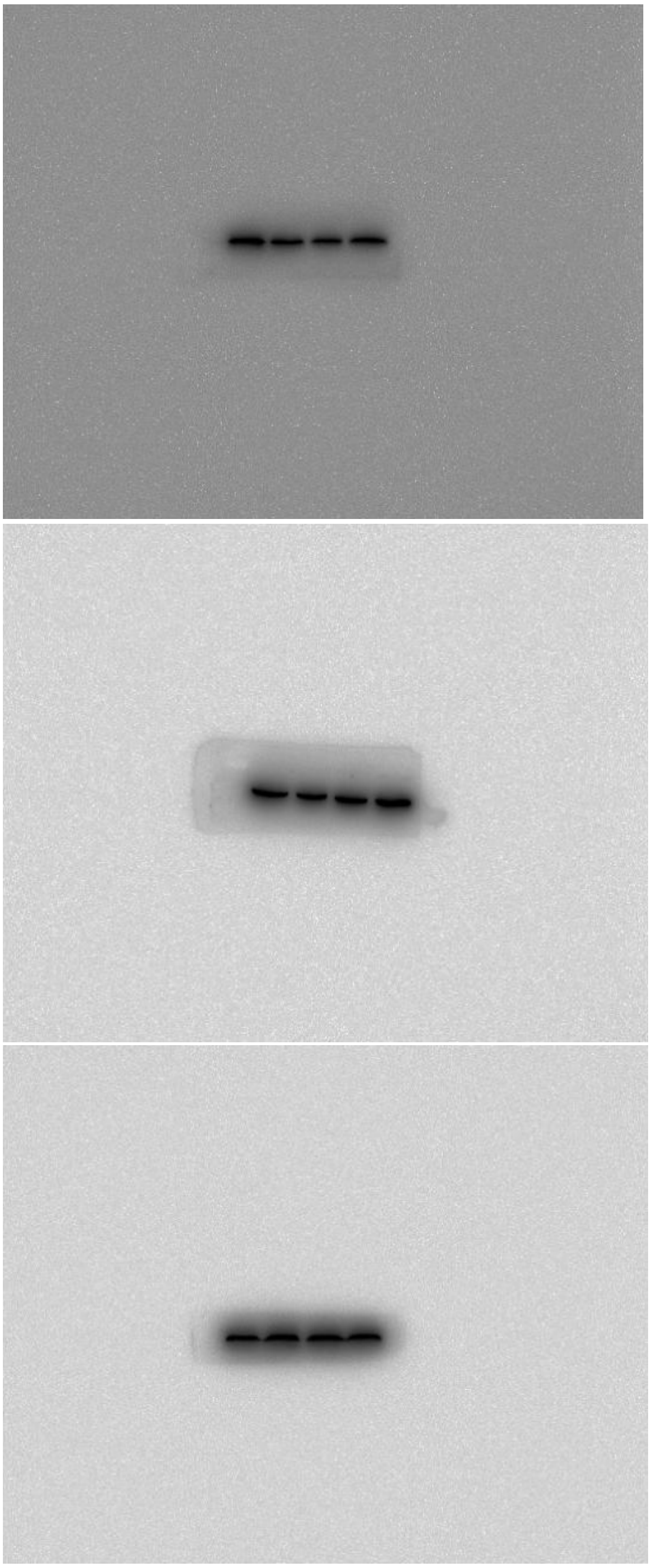

Figure 5C

MMP2

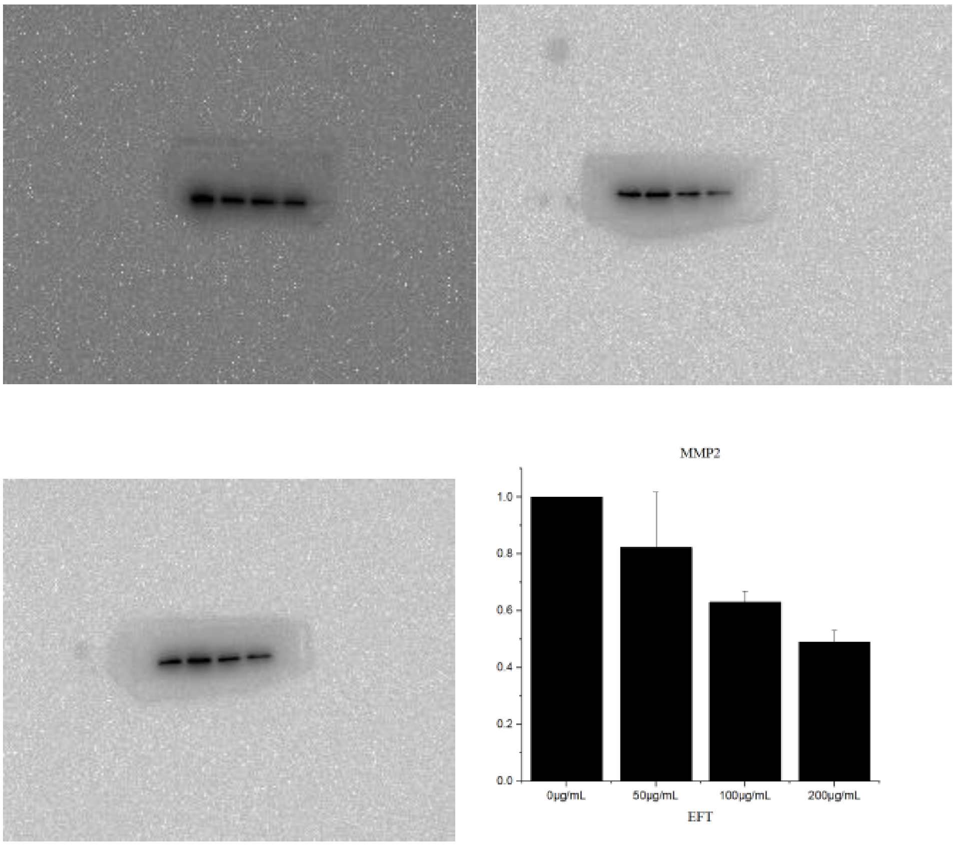

Figure 5C

MMP9

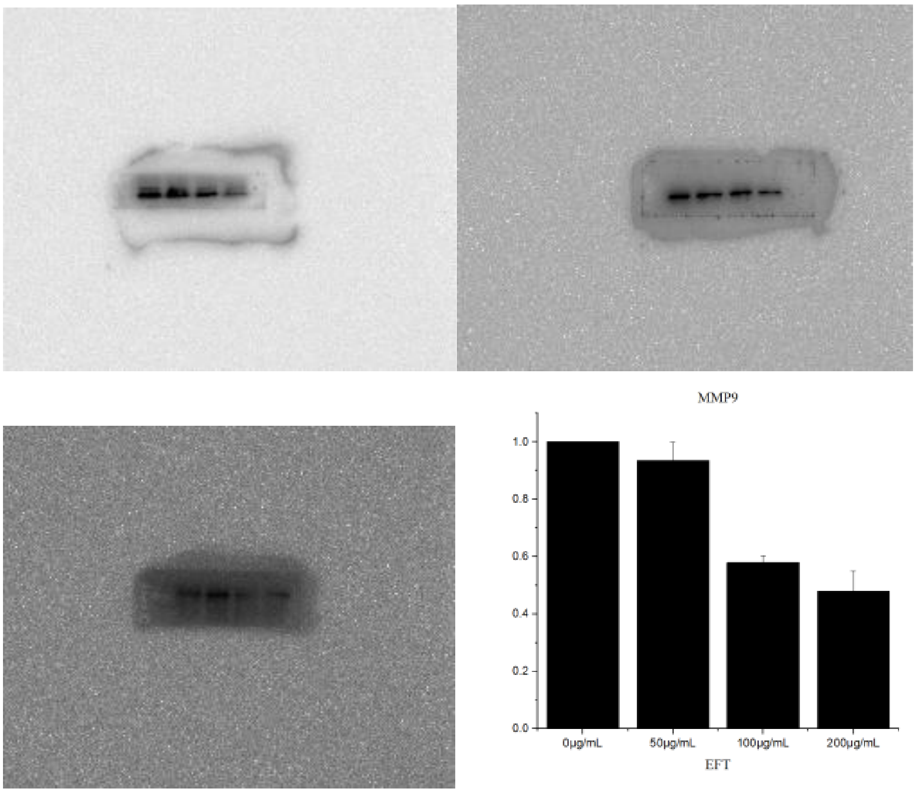

Figure 5C

β-actin

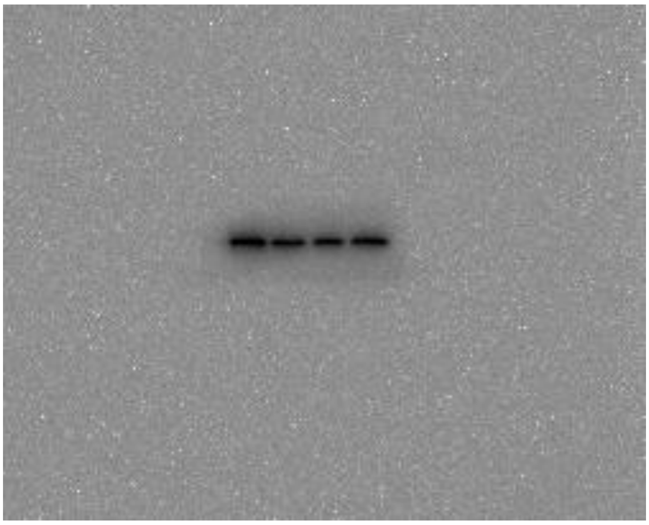

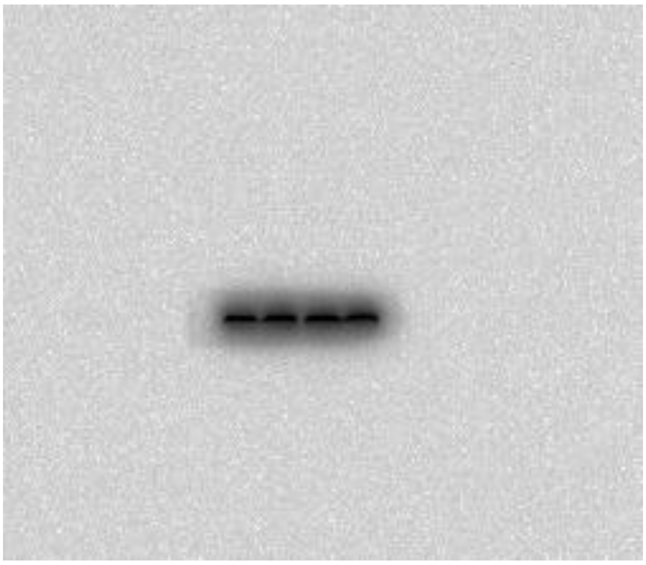

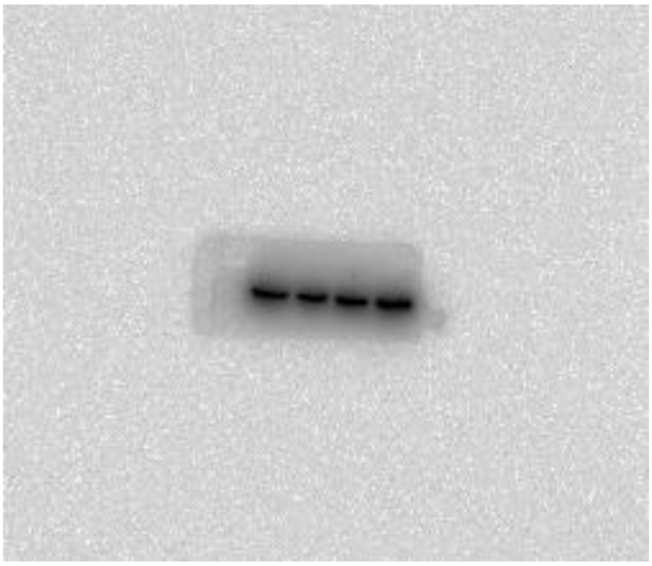

Method:

The lysates were boiled for 5 min at 100 ℃. Equal amounts of total protein were separated by 8%–10% sodium dodecyl sulfate-polyacrylamide gel electrophoresis (SDS-PAGE), then transferred to PVDF, blocked with 5 % non-fatty milk in TBS-Tween buffer (pH 7.5, 0.12 M Tris-base, 1.5 M NaCl, 0.1 % Tween-20) for 2 h at room temperature. PVDF membrane was cut into smaller membrane pieces according to the different molecular weights of the targeted proteins. The cropped PVDF membrane was incubated separately with the corresponding primary antibody at 4℃ overnight. Then incubated with HRP conjugated secondary antibodies (goat-antirabbit or goat-antimouse) at room temperature in the dark for 2 h. Detection was performed using an ECL system. Protein quantification was normalized to β-actin expression. ImageJ software was used to quantify band intensities.

Additional information:

We have submitted the original gel images as we could. However, we didn't retain the full gel images as we used to test the targeted proteins and reference proteins at the same time (the blots cut prior to hybridisation with antibodies). But the cropped gels we submitted were the original ones which can see the important bands or the boundary of the gels. And we will keep the full gel images in our next work.
